# Viral RNA *N*^6^-methyladenosine modification modulates both innate and adaptive immune responses of human respiratory syncytial virus

**DOI:** 10.1371/journal.ppat.1010142

**Published:** 2021-12-20

**Authors:** Miaoge Xue, Yuexiu Zhang, Haitao Wang, Elizabeth L. Kairis, Mijia Lu, Sadeem Ahmad, Zayed Attia, Olivia Harder, Zijie Zhang, Jiangbo Wei, Phylip Chen, Youling Gao, Mark E. Peeples, Amit Sharma, Prosper Boyaka, Chuan He, Sun Hur, Stefan Niewiesk, Jianrong Li

**Affiliations:** 1 Department of Veterinary Biosciences, College of Veterinary Medicine, The Ohio State University, Columbus, Ohio, United States of America; 2 Department of Biological Chemistry and Molecular Pharmacology, Harvard Medical School, Boston, Massachusetts, United States of America; 3 Program in Cellular and Molecular Medicine, Boston Children’s Hospital, Boston, Massachusetts, United States of America; 4 Department of Chemistry, Department of Biochemistry and Molecular Biology, and Institute for Biophysical Dynamics, The University of Chicago, Chicago, Illinois, United States of America; 5 Center for Vaccines and Immunity, Abigail Wexner Research Institute at Nationwide Children’s Hospital, Columbus, Ohio, United States of America; 6 Department of Pediatrics, The Ohio State University College of Medicine, Columbus, Ohio, United States of America; 7 Howard Hughes Medical Institute, The University of Chicago, Chicago, Illinois, United States of America; 8 Howard Hughes Medical Institute, Boston Children’s Hospital, Boston, Massachusetts, United States of America; Duke University Medical Center, UNITED STATES

## Abstract

Human respiratory syncytial virus (RSV) is the leading cause of respiratory tract infections in humans. A well-known challenge in the development of a live attenuated RSV vaccine is that interferon (IFN)-mediated antiviral responses are strongly suppressed by RSV nonstructural proteins which, in turn, dampens the subsequent adaptive immune responses. Here, we discovered a novel strategy to enhance innate and adaptive immunity to RSV infection. Specifically, we found that recombinant RSVs deficient in viral RNA *N*^6^-methyladenosine (m^6^A) and RSV grown in m^6^A methyltransferase (METTL3)-knockdown cells induce higher expression of RIG-I, bind more efficiently to RIG-I, and enhance RIG-I ubiquitination and IRF3 phosphorylation compared to wild-type virion RNA, leading to enhanced type I IFN production. Importantly, these m^6^A-deficient RSV mutants also induce a stronger IFN response in vivo, are significantly attenuated, induce higher neutralizing antibody and T cell immune responses in mice and provide complete protection against RSV challenge in cotton rats. Collectively, our results demonstrate that inhibition of RSV RNA m^6^A methylation enhances innate immune responses which in turn promote adaptive immunity.

## Author summary

RSV is the most important cause of upper and lower respiratory tract infection of infants, young children, immunocompromised individuals, and the elderly. Despite major efforts, no vaccine is available for RSV. A live attenuated vaccine is one of the most promising vaccine approaches for RSV. However, a major problem is that natural RSV infection does not induce long-lived protection. The reason likely lies in its NS1 and NS2 proteins’ strong inhibition of type I interferon (IFN) induction, which impairs subsequent adaptive immunity. We found that RSV RNA contains *N*^6^-methyladenosine (m^6^A) and that recombinant RSVs lacking m^6^A methylation induce significantly higher type I IFN in cell culture and in mice compared to the parental RSV. Despite the fact that these m^6^A-deficient RSVs are significantly attenuated, they induce higher RSV-specific antibody and T cell immune responses in mice and provide complete protection against RSV challenge in cotton rats. Thus, m^6^A-deficient RSVs are more immunogenic than the parental RSV, highlighting that suppression of viral RNA m^6^A methylation is a novel strategy to enhance innate immunity which, in turn, enhances adaptive immunity.

## Introduction

Human respiratory syncytial virus (RSV) is a major cause of respiratory tract infections in humans. RSV infects individuals of all ages, with high morbidity and mortality seen in infants, children, elders and immunocompromised individuals. RSV is the most important cause of pediatric respiratory tract infection, and the second most important cause of respiratory infection in elders, second only to influenza virus [1]. The clinical signs and symptoms associated with RSV range from mild respiratory problems to severe cough, bronchiolitis, pneumonia, and death. Worldwide it is estimated that RSV causes 3.4 million hospitalizations and between 66,000 and 199,000 deaths in young children less than 5 years of age [2,3]. Although RSV was first discovered in 1953, there is no FDA-approved vaccine or antiviral drug treatment. In the 1960s, a formalin-inactivated (FI) RSV vaccine candidate was developed and tested in human clinical trials. This vaccine candidate not only failed to induce protection but led to an enhanced respiratory disease (ERD) upon RSV infection [4,5]. In recent years, many different RSV vaccine candidate types have been developed, including live attenuated, subunit, mRNA, virus-like particles, and viral vectored vaccines [6–8]. Among these, live attenuated vaccines are one of the most promising candidates [6,7,9].

One of the major challenges in the development of a live attenuated RSV vaccine is how to enhance the innate immune response to an attenuated RSV strain [10–12]. A robust innate immune response is likely essential for inducing a strong and durable adaptive immune response [11,12]. It is known that humans can be repeatedly infected by RSV during their life time, which is in part due to the fact that RSV infection strongly inhibits innate immunity, leading to a blunted adaptive immune response by neutralizing antibody and memory B- and T-cells [10–13]. In animal models and human clinical trials, several live vaccine candidates were found to lack sufficient immunogenicity and/or fail to induce long-lasting neutralizing antibodies and protection [12,14,15].

Current efforts to enhance RSV innate immunity are exclusively focused on the two RSV nonstructural proteins (NS1 and NS2), which were shown to strongly impair type I interferon (IFN) production by interfering with recognition by RIG-I or by promoting proteasomal degradation of STAT2 [16–19]. In fact, RSV lacking NS1 (ΔNS1) and/or NS2 (ΔNS2) have been generated and tested in animal models [20–22]. The ΔNS1 virus induced more type I IFN and was immunogenic in chimpanzees, in addition to being highly attenuated [20]. However, this virus grows poorly even in Vero cells where interferon is not produced which would make large-scale production of this vaccine candidate difficult [16,20]. ΔNS2 virus grows to a relatively high titer but is less attenuated in chimpanzees. Deletion of ΔNS2 is not sufficient to remove the inhibitory effect of innate immune response because ΔNS2 virus still expresses the NS1 protein, the major suppressor of type I IFN response [23]. Currently, it is unclear whether these recombinant viruses are robust enough to induce a strong innate immunity and long-lasting adaptive immunity against RSV infection. Therefore, exploration of other strategies to enhance the innate immunity of a live attenuated RSV vaccine are urgently needed.

Among more than 160 RNA modifications, *N*^6^-methyladenosine (m^6^A) is the most abundant internal modification in mRNA [24]. It is present in many different eukaryotic species including yeast, plants, flies and mammals, contributing to fundamental functions such as RNA metabolism, transportation and translation [25,26]. Host “writer” proteins, METTL3 (a catalytic subunit) and METTL14 (an allosteric activator), catalyze m^6^A methylation, which can be removed by m^6^A “eraser” proteins FTO and ALKBH5 [27,28]. Biological functions of m^6^A are mediated through m^6^A “reader” proteins including YTHDF1, YTHDF2, YTHDF3, and YTHDC1. In the early 1970s, it was found that RNAs produced by several DNA and RNA viruses contained m^6^A methylation [29–31]. However, recently it was found that viral m^6^A methylation can either play a positive or negative role in viral replication and gene expression, depending on the specific virus and/or the cell type [32–36]. We recently mapped m^6^A sites in the genome, antigenome, and mRNAs of two pneumoviruses [RSV and human metapneumovirus (hMPV)] [37,38]. For both viruses, the G gene, which is located in the middle of genome, contains the highest number of methylated m^6^A sites. In addition, viral m^6^A methylation promotes replication and gene expression of RSV and hMPV [37,38].

During virus infection, the host innate immune system must discriminate viral (non-self) from cellular (self) nucleic acids to mount a protective immune response when appropriate. On the other hand, viruses often mask their nucleic acids with chemical modifications in order to escape detection by host innate immune surveillance. Short synthetic RNAs with chemical modifications including m^6^A, m^5^C, m^5^U, s^2^U or Ψ suppress Toll-like receptor 3 (TLR3), TLR7 and TLR8 signaling [39,40]. Short synthetic RNAs derived from the 3′ untranslated region of hepatitis C virus containing m^6^A methylation inhibit the RIG-I signaling pathway [41]. Thus, viral RNA lacking posttranscriptional modifications is likely recognized by host innate immunity as non-self RNAs, inducing a stronger type I IFN response in order to restrict virus replication and spread. Recently, we provided the first evidence that hMPV lacking m^6^A methylation induced significantly more type I IFN and that m^6^A methylation serves as a molecular marker for the host to discriminate self RNA from non-self RNA [38]. However, whether viral m^6^A methylation can modulate adaptive immunity is unknown.

Here, we found that viral m^6^A methylation not only modulates innate immunity but also adaptive immunity during RSV infection. We generated m^6^A-deficient RSV by growing RSV in a cell line lacking METTL3, the major catalytic subunit of m^6^A methyltransferase, or by synonymous mutations in m^6^A sites of the viral G gene, which contains the most abundant m^6^A peaks. These m^6^A-deficient viruses or virion RNAs induced significantly higher type I IFN responses than wt virus or m^6^A-sufficient virion RNA. Mechanistically, m^6^A-deficient virion RNA binds more efficiently to RIG-I, induces higher expression of RIG-I, and enhances RIG-I ubiquitination and IRF3 phosphorylation, leading to enhanced RIG-I mediated IFN signaling. Importantly, we found that m^6^A-deficient RSV mutants induce higher IFN responses, more neutralizing antibody, and T cell immune responses in mice compared to the parental recombinant green fluorescent protein expressing RSV (rgRSV). In addition, cotton rats immunized with these m^6^A-deficient RSVs were completely protected from RSV challenge. Our results demonstrate that viral m^6^A methylation modulates both innate and adaptive immunity and that inhibition of viral m^6^A methylation represents a novel strategy to improve the efficacy of RNA live attenuated vaccine candidates.

## Results

### Virion RNA purified from RSV grown in *METTL3*-knockdown cells is defective in m^6^A methylation

**Fig 1:**
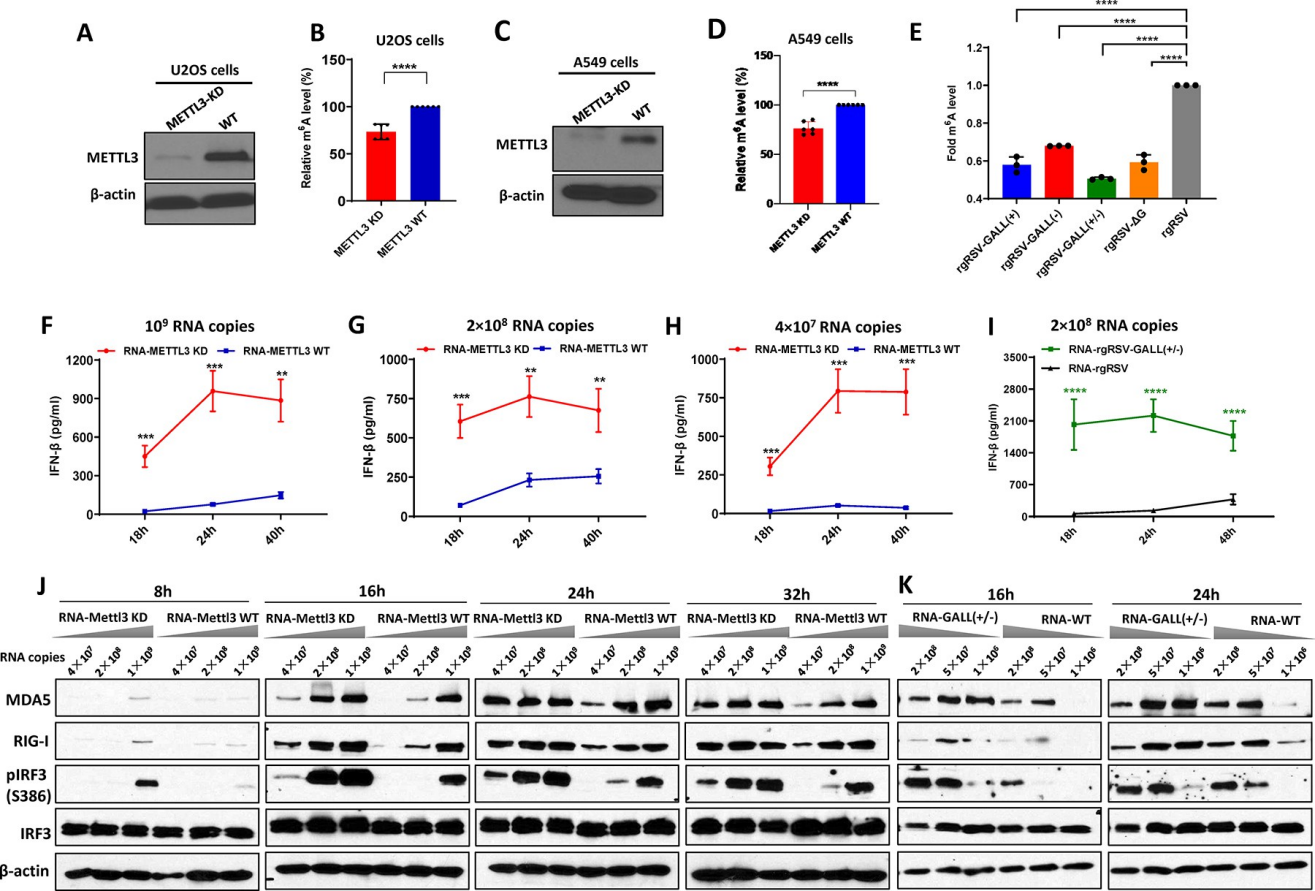
m^6^A-deficient virion RNAs induce higher type I IFN responses in cell culture. (A) Western blot showing METTL3 expression in *METTL3*-knockdown (KD) U2OS cells and wild type U2OS cells. (B) Relative m^6^A level in virion RNA from RSV grown on *METTL3* KD U2OS cells and wild type (wt) U2OS cells. The content of m^6^A level for each virion RNA was quantified by m^6^A methylation kit. (C) Western blot showing METTL3 expression in *METTL3*-KD A549 cells and control sgRNA transduced A549 cells. (D) Relative m^6^A level in virion RNA from RSV grown on *METTL3*-KD A549 cells and control sgRNA transduced A549 cells. (E) Relative m^6^A level in virion RNA from rgRSV mutants and wt rgRSV. rgRSV with G gene deletion mutant (rgRSV-ΔG), rgRSV-GALL(+), rgRSV-GALL(-), rgRSV-GALL(+/-), and rgRSV grew in HEp-2 cells and purified. The content of m^6^A level for each virion RNA was quantified. (F-H) A549 cells were transfected with 10^9^ RNA copies (F), 2×10^8^ RNA copies (G) and 4×10^7^ RNA copies (H) of virion RNA of RSV grown on *METTL3*-KD or WT cells, IFN-β was measured at indicated time points. (I) Comparison of IFN response virion RNA of rgRSV-GALL(+/-) and WT rgRSV. A549 cells were transfected with of 2×10^8^ RNA copies of mutant virus and IFN-β was measured. (J and K) m^6^A-deficient RSV RNA increases expression of RIG-I and MDA5 and induces higher IRF3 phosphorylation. A549 cells were transfected with virion RNA of RSV grown on *METTL3* KD/WT cells (J) at dose of 1.0×10^9^, 2×10^8^ and 4×10^7^ RNA copies, or virion RNA of rgRSV-GALL(+/-) and parental rgRSV (K) at dose of 2×10^8^, 5×10^7^, and 1×10^6^ RNA copies. At indicated times, cell lysates were analyzed by Western blotting using antibodies specific to RIG-I, MDA5, IRF3, IRF3 (phosphorylated at S386) or β-actin. Data shown are mean ± s.d. of *n* =  6 (B, D) or *n* =  3 (E-I) independent experiments. Western blots are representative of *n* =  3 biologically independent experiments. Statistical significance was determined by two-sided Student’s *t*-test. *P < 0.05; **P < 0.01; ***P < 0.001; ****P < 0.0001.

To begin to explore the role of viral m^6^A methylation in innate and adaptive immunity, we first generated RSV RNA that was naturally defective in m^6^A methylation using METTL3 knockdown (KD) U2OS cells. The m^6^A level of viral RNA will likely be reduced when virus grows in cells with depletion of *METTL3*, the catalytic subunit of host m^6^A methyltransferase. Western blot confirmed that only trace amounts of METTL3 were detected in *METTL3*-KD U2OS cells (**Fig 1A**). Then, rgRSV was grown in wild type (wt) U2OS and *METTL3*-KD U2OS cells and rgRSV virions were purified from supernatants by sucrose gradient ultracentrifugation. Virion RNA was extracted and quantified by real-time RT-PCR. The m^6^A level in RSV RNA was measured by a commercial RNA m^6^A methylation kit. As shown in **Fig 1B**, RSV RNA purified from virions grown in *METTL3*-KD U2OS cells had approximately 27% reduction in m^6^A content compared to virion RNA purified from virions grown in wt U2OS cells (****P<0.0001). However, virion RNA from *METTL3*-KD U2OS cells was not completely defective in m^6^A methylation, which is likely due to the incomplete elimination of METTL3 (**Fig 1A**) and/or the existence of other m^6^A methyltransferases in host cells [42]. In fact, other host methyltransferases (e.g. METTL16) and cofactors (e.g. WTAP) can catalyze m^6^A methylation and/or modulate m^6^A methylation [42–44]. Since the abundance of m^6^A methyltransferase METTL3 is variable in different cell lines [45], we compared the percent of m^6^A reduction of virion RNA purified from *METTL3*-KD A549 cells and control sgRNA treated A549 cells, an adenocarcinomic human alveolar basal epithelial cell line.

Although we aimed to generate *METTL3*-knockout (KO) A549 cells by CRISPR/Cas9 technique, the single cell clone we obtained contained a trace amount of METTL3 protein detected by Western blot (**Fig 1C**). Thus, this cell line was named *METTL3*-KD A549 cells. Similar to *METTL3*-KD U2OS cells, RSV RNA purified from virions grown in *METTL3*-KD A549 cells had approximately 24% reduction in m^6^A content compared to virion RNA purified from virions grown in control A549 cells (****P<0.0001) (**Fig 1D**). Thus, RSV RNA purified from virions grown in *METTL3*-KD cells was defective in m^6^A methylation without altering its nucleotide sequence.

### Recovery of m^6^A-deficient rgRSV mutants by removal of m^6^A sites in the viral G gene

Previously, we performed m^6^A-seq for RSV RNA and found that the RSV genome, antigenome, and mRNAs were m^6^A modified [37]. Interestingly, the G gene in the genome, the G gene region in the antigenome, and the G mRNA contain the highest modified m^6^A site. Using synonymous mutations, we previously recovered an m^6^A-deficient RSV mutant (rgRSV-G123) in which all putative m^6^A sites in the G gene region in the positive-sense RNA (antigenome and mRNA) were removed [37]. As expected, rgRSV-G123 was defective in m^6^A in the antigenome and G mRNA but not in the genome. The rgRSV-G123 m^6^A mutant was renamed rgRSV-GALL(+) (**S1 Fig**). Here, we recovered two more m^6^A-deficient rgRSV mutants from an infectious RSV cDNA clone. The first rgRSV mutant is rgRSV-GALL(-), in which all the putative m^6^A sites on the G gene in the RSV genome were removed using synonymous mutations (**S2 Fig**). The second rgRSV mutant is rgRSV-GALL(+/-), which is the combination of all the m^6^A sites mutations from both the G region of RSV genome and the G gene region of antigenome/mRNA. All recombinant viruses were purified, virion RNA was extracted, and the level of m^6^A was measured with the m^6^A quantification kit. As shown in **Fig 1E**, the m^6^A level in virion RNA of rgRSV-GALL(+), rgRSV-GALL(-) and rgRSV-GALL(+/-) were reduced by 30% to 50% (*P*<0.05) compared to the virion RNA of the parental rgRSV. As expected, the combined mutant rgRSV-GALL(+/-) was more defective in m^6^A level than rgRSV-GALL(+) or rgRSV-GALL(-). In this experiment, we also used rgRSV-ΔG which lacks the G gene as a control. We found that the m^6^A level of each rgRSV mutant was similar to that of rgRSV-ΔG, consistent with our earlier finding that most of the m^6^A modifications in the rgRSV genome are within the G region of the RSV genome and antigenome [46]. Thus, these experiments demonstrated that rgRSV-GALL(+), rgRSV-GALL(-) and rgRSV-GALL(+/-) genome RNAs were indeed defective in m^6^A methylation.

### m^6^A-deficient virion RNA induces a higher type I IFN response

Having generated a panel of m^6^A-deficient virion RNAs either by purification from virions grown in *METTL3*-KD cells or by site-directed synonymous mutagenesis, we next tested their abilities to trigger type I IFN by transfection of virion RNA into A549 cells. Under these conditions, without viral proteins, there would be no RNA replication or viral protein synthesis, thereby eliminating any effects of viral proteins on IFN signaling. Briefly, the same amount of virion RNA from each virus was transfected into A549 cells, and IFN-β was measured at different times. Interestingly, RNA purified from virions grown in *METTL3*-KD U2OS cells induced significantly more IFN-β than RNA purified from virions grown in wt U2OS cells at 18, 24, and 40 h post-transfection at all three RNA doses (**Fig 1F, 1G, and 1H**). Similarly, rgRSV-GALL(+/-) virion RNA induced significantly more IFN-β than that of rgRSV at 18, 24, and 48 h after transfection (**Fig 1I**). Therefore, m^6^A-deficient RSV virion RNA induced significantly more type I IFN than m^6^A-sufficient RNA.

### m^6^A-deficient virion RNA induces a stronger type I IFN response pathway

For cytoplasmic replicating RNA viruses, the invading viral RNA is detected by RIG-I-like receptors (RLRs) including RIG-I and MDA5 [47,48]. Upon recognition, RIG-I and MDA5 undergo a significant conformational change that transmits a signal to the downstream adaptor protein MAVS, activating IRF3 phosphorylation and translocation to the nucleus, leading to IFN production. To further investigate the mechanism underlying the enhanced IFN response associated with m^6^A-deficient virion RNA, we examined the expression of molecules involved in the response to type I IFN. Briefly, A549 cells were transfected with equivalent amounts of virion RNA from m^6^A-deficient viruses or parental rgRSV at increasing doses, and the expression of RIG-I, MDA5, IRF3, and phosphorylated IRF3 was detected by Western blot. RNA purified from RSV virions grown in *METTL3*-KD U2OS cells induced significantly higher expression of RIG-I, MDA5, and IRF3 phosphorylation than RNA purified from virions grown in wt U2OS cells (**Fig 1J**). The difference was more obvious when a lower dose of RNA was used for transfection. The peak MDA5, RIG-I, and pIRF3 expression appeared around 16–24 h post-transfection. When the incubation time was extended to 48 and 72h, we found that expression of MDA5 and pIRF3 was reduced (**S3 Fig**) and cells became unhealthy, probably due to the apoptosis induced by the high activation of IFN [49]. Similarly, virion RNA of rgRSV-GALL(+/-) induced higher levels of RIG-I, MDA5, and IRF3 phosphorylation (**Fig 1K**) compared to virion RNA of the parental rgRSV. Collectively, these results demonstrated that m^6^A-deficient virion RNA activated the type I IFN signaling pathway higher than m^6^A-sufficient virion RNA. Since both RIG-I and MDA5 belong to the interferon-stimulated gene (ISG) family, the increase in IFN level by m^6^A-deficient RSV RNA likely stimulates the expression of ISGs including RIG-I and MDA5.

### RIG-I is the major RNA sensor that recognizes m^6^A-deficient RSV RNA

**Fig 2:**
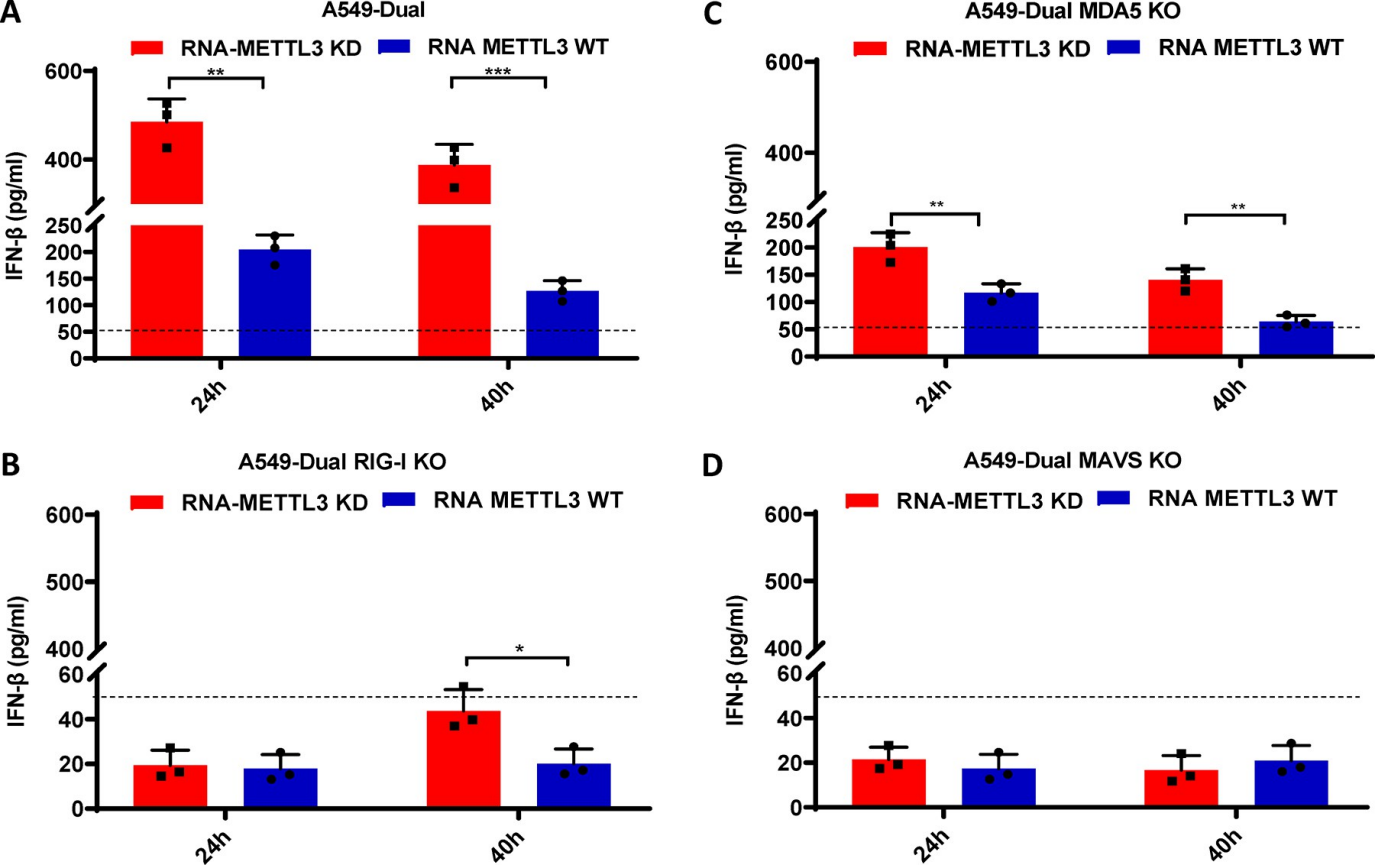
RIG-I is the main RNA sensor that recognizes m^6^A-deficient RSV virion RNA. Confluent wild-type (A), *RIG-I*-knockout (B), *MDA5*-knockout (C), or *MAVS*-knockout (D) A549 Dual cells were transfected with same amount (2×10^7^ RNA copies) of RSV virion RNAs from either *METTL3* KD cells or *METTL3* WT cells. Cell culture supernatants were harvested at 24 and 40 h after inoculation, IFN-β in the supernatant at indicated time points was measured by commercial ELISA kit. Error bars represent SD from *n*  =  3 independent experiments. *P<0.5, **P < 0.01, ***P < 0.001, ****P < 0.0001.

To directly determine which RNA sensors are involved in the detection of m^6^A-deficient RSV RNA, we examined the IFN response of m^6^A-deficient virion RNA in A549 cells lacking RIG-I, MDA5, or their downstream adaptor protein, MAVS. Briefly, wt A549-Dual or KO A549-Dual cells were transfected with RNA from virions grown in *METTL3*-KD U2OS cells or wt U2OS cells, and IFN-β was quantified by ELISA. As shown in **Fig 2A**, RSV virion RNA from *METTL3*-KD U2OS cells triggered significantly more IFN-β production than virion RNAs from wt U2OS cells in wt A549-Dual cells (*P* < 0.05; Student’s *t*-test). Notably, when RIG-I was knocked out, IFN-β level was below the detection limit (50 pg/ml) at both 24 and 40 h post-transfection (**Fig 2B**). In contrast, a considerable level of IFN-β was still detectable in *MDA5*-KO A549-Dual cells (**Fig 2C**) although there was a significant reduction compared to the wt A549-Dual cells. In addition, RSV virion RNA from *METTL3*-KD U2OS cells triggered significantly more IFN-β production than virion RNAs from wt U2OS cells in *MDA5*-KO A549-Dual cells. IFN-β production was completely abrogated in A549-Dual cells lacking MAVS **(Fig 2D)**. These results demonstrate that RIG-I plays a major role and MDA5 plays a minor role in recognizing m^6^A-deficient RSV RNA.

### Enhanced recognition of m^6^A-deficient viral RNA by RIG-I

To further explore the mechanisms by which m^6^A inhibits the innate immune response to viral RNA, we directly compared the binding affinity of m^6^A-sufficient and m^6^A-deficient viral RNA to RIG-I protein as previously described [50]. Briefly, the same amount of each viral RNA was incubated with cell lysate containing Flag-tagged RIG-I protein. The viral RNA pulled down by RIG-I was quantified by real-time PCR by using primer pairs that anneal to the genome or antigenome. As the quantity control, the amount of RIG-I protein in each sample was equivalent, confirming that the pulldown efficiency of each group was similar (**S4A and S4B Fig**). As shown in **S4C and S4D Fig**, RIG-I pulled down significantly more virion RNA from rgRSV-GALL(+/-) than that from rgRSV. In this case, a 4-fold and 3-fold increase in RIG-I binding was observed for antigenome and genome, respectively. Similarly, RIG-I pulled down significantly more RSV virion RNA (genome and antigenome) from *METTL3*-KD U2OS cells than RSV virion RNA from wt U2OS cells (**S4E and S4F Fig**). A 50-fold and 17-fold increase in RIG-I binding was observed for m^6^A-deficient antigenome and genome, respectively (**S4E and S4F Fig**). These results indicate that m^6^A-deficiency enhances RSV RNA binding to RIG-I.

### m^6^A-deficient RSV RNA enhances RIG-I ubiquitination

**Fig 3:**
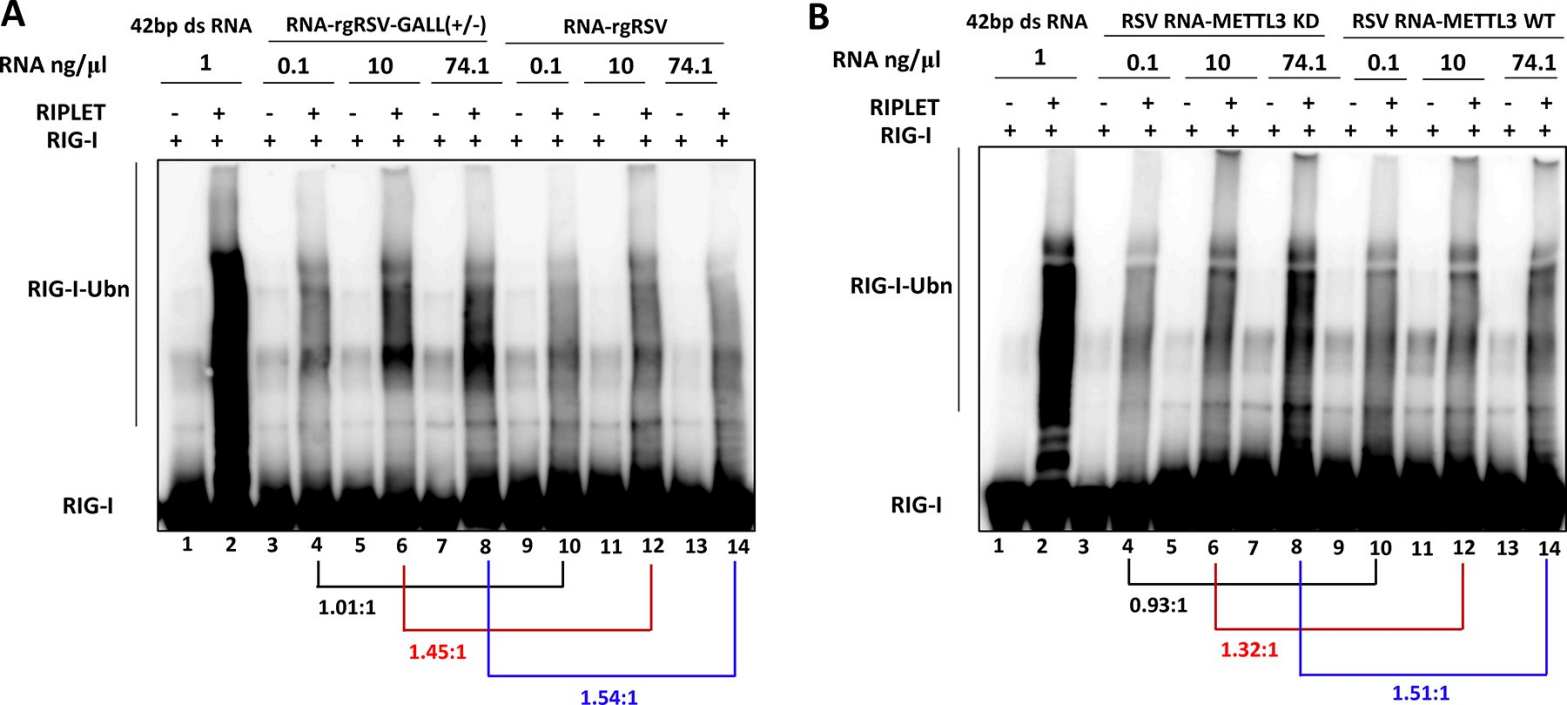
In vitro ubiquitination analysis of RIG-I. (A) rgRSV-GALL(+/-) virion RNA enhances RIG-I ubiquitination (RIG-I-Ubn) compared to rgRSV virion RNA. 1.0 μM of purified RIG-I was incubated with 1ng/ μl of 42 bp dsRNA or different doses (0.1, 10, and 74.1 ng/ μl) of virion RNA from rgRSV-GALL(+/-) or parental rgRSV with or without RIPLET. Ubiquitination of RIG-I was analyzed by anti-RIG-I blot. (B) Virion RNA from *METTL3*-KD U2OS cells enhances RIG-I ubiquitination compared to virion RNA from wt U2OS cells. The density of each lane was quantified by Image J. The length of lane used for quantification was indicated by the line in the left side. The ratio between m^6^A-deficient virion RNA and wild type virion RNA at each RNA concentration is indicated.

Ubiquitination plays essential roles in RIG-I activation as well as the type I IFN signaling pathway. Upon binding to the RNA ligand, RIG-I undergoes a significant conformational change enabling K63-linked ubiquitination at multiple sites that is required to reach full activity. Recent studies have found that RIG-I ubiquitination and activation are dependent on RIPLET [51]. Thus, we compared the abilities of m^6^A-deficient and sufficient RSV RNA in promoting ubiquitination of RIG-I. In this experiment, a 42bp dsRNA was used as a positive control. As expected, incubation of purified RIG-I with 42bp dsRNA triggered strong ubiquitination of RIG-I in the presence of RIPLET (**Fig 3A, lane 2**). No RIG-I ubiquitination was observed in the absence of RIPLET (**Fig 3A, lane 1**). As shown in **Fig 3A and 3B**, RSV RNA promoted RIG-I ubiquitination in a dose-dependent manner only in the presence of RIPLET. The ratio of RIG-I ubiquitination between m^6^A-deficient RSV RNA and WT-RNA was quantified by image J. At an RNA concentration of 0.1 ng/μl, the ratio of RIG-I ubiquitination between rgRSV-GALL(+/-) RNA and WT rgRSV RNA is approximately 1.01: 1 (**Fig 3A**). However, a 1.45- and 1.54-fold increase in RIG-I ubiquitination occurred at rgRSV-GALL(+/-) RNA concentrations of 10 and 74.1 ng/μl, respectively (**Fig 3A, compare lanes 6 and 12, and lanes 8 and 14**). Similarly, virion RNA from *METTL3*-KD U2OS cells induced 1.32 and 1.51-fold increases in RIG-I ubiquitination at RNA concentrations of 10 and 74.1 ng/μl, respectively, compared to virion RNA from wt U2OS cells (**Fig 3B, compare lanes 6 and 12, and lanes 8 and 14**). To further confirm these results, a separate ubiquitination assay was carried out using RNA concentrations ranging from 2 to 150 ng/μl (**S5 Fig**). Similarly, there was no difference in RIG-I ubiquitination at a relatively low dose of RSV RNA (2 ng/μl) (**S5A Fig**). A 1.38–1.90 fold increase in RIG-I ubiquitination was observed for RNA from *METTL3*-KD cells (**S5B Fig**) and rgRSV-GALL(+/-) RNA (**S5C Fig**) at concentration of 50–150 ng/μl compared to WT RNA. These data demonstrate that m^6^A-deficient RSV RNA promotes RIG-I ubiquitination.

### m^6^A-deficient rgRSV infection triggers higher IFN production in vitro

Having demonstrated that m^6^A-deficient RSV virion RNA enhanced the IFN response, we next examined whether m^6^A-deficient RSV infection can directly induce a higher type I interferon response. It should be noted that the IFN response is more complicated during virus infection as RNA species produced during replication induce IFN production while the RSV nonstructural proteins, NS1 and NS2, strongly inhibit IFN responses.

**Fig 4:**
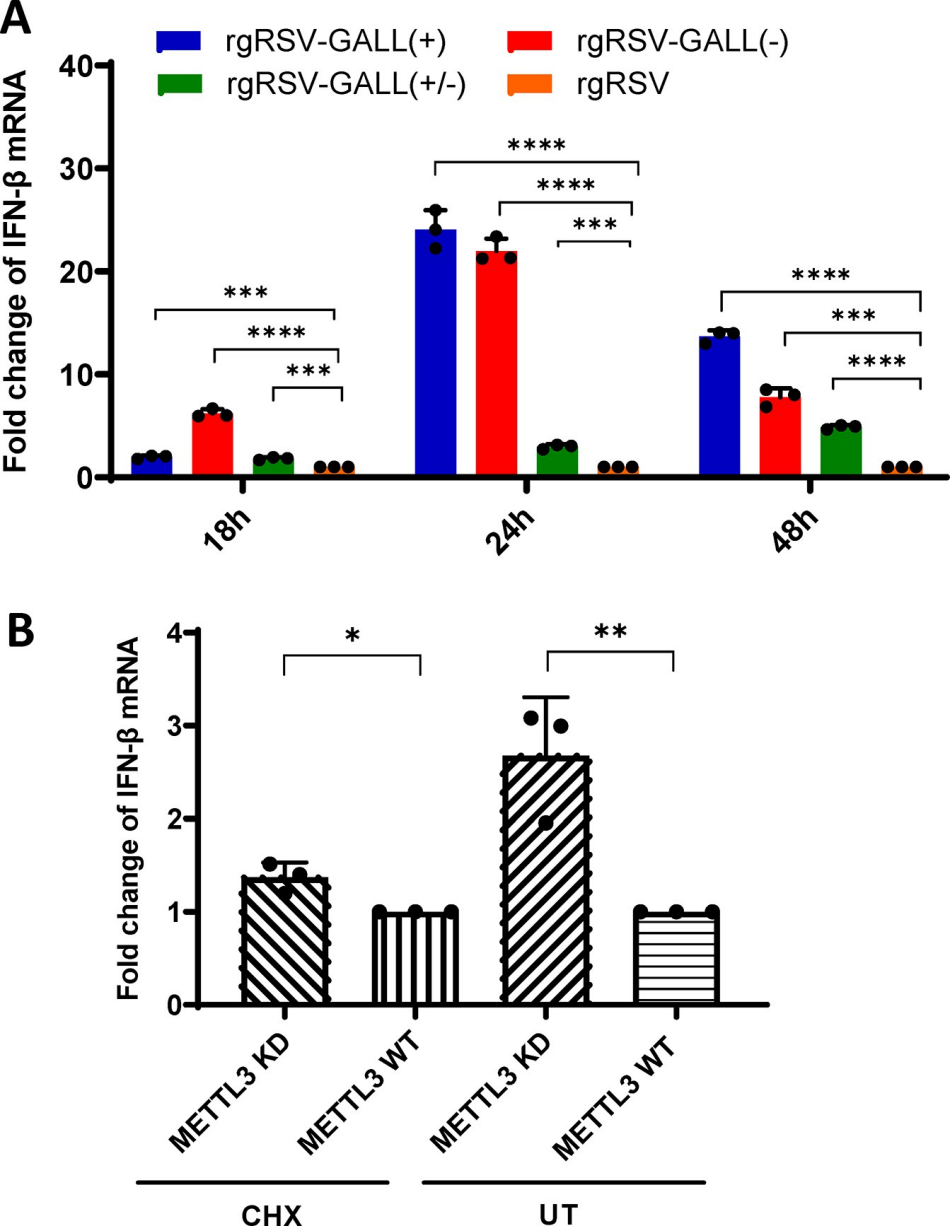
IFN response in A549 cell after RSV infection. (A) IFN-β mRNA level in A549 cells infected by rgRSV mutants or rgRSV. A549 cells were infected by rgRSV-GALL(+), rgRSV-GALL(-), rgRSV-GALL(+/-) or rgRSV at an MOI of 0.5. At 18, 24, and 48 h post-infection, total RNA was harvested and IFN-β mRNA in cells was quantified by real-time RT-PCR. β-actin was used as an internal control. Error bars represent SD from *n*  =  3 independent experiments. (B) IFN-β mRNA level in A549 cells infected by rgRSV grown from *METTL3*-KD U2OS or WT U2OS cells with or without CHX treatment. A549 cells were treated for 1 h with 0 (UT, untreated) or 50 μg/ml CHX, and then infected either with rgRSV grown from *METTL3*-KD U2OS or WT U2OS cells at MOI of 0.5. At 24 h post-infection, total RNA was extracted and IFN-β mRNA was quantified by real-time RT-PCR. β-actin was used as an internal control. Error bars represent SD from *n*  =  3 independent experiments. *P<0.5, **P < 0.01, ***P < 0.001, ****P < 0.0001.

Here we examined A549 cells infected by rgRSV mutants for their induction of IFN-β mRNA. Briefly, confluent A549 cells were infected with rgRSV-GALL(+), rgRSV-GALL(-), rgRSV-GALL(+/-) or parental rgRSV at an MOI of 0.5. Cell lysates were harvested at 18, 24, and 48h post-infection, total RNA was extracted, and IFN-β mRNA was quantified by real-time RT-PCR. All m^6^A-deficient rgRSV mutants triggered significantly more IFN-β mRNA than the parental rgRSV at each time point (**Fig 4A**). Taken together, these results indicate that m^6^A-deficient rgRSVs induce higher type I IFN than the parental rgRSVs during viral infection.

We examined the ability of rgRSV that was m^6^A-deficient because they were grown in *METTL3*-KD U2OS cells to induce an IFN response by limiting replication to the first step in virus replication, primary transcription. Once the virus genome begins to replicate in A549 cells, it would be m^6^A modified. Briefly, A549 cells were infected in the presence or absence of cycloheximide (CHX) with rgRSV grown in wt or *METTL3*-KD U2OS cells. CHX allows virus attachment, entry of nucleocapsids, and primary transcription, but not genome replication which requires ongoing viral protein synthesis [52]. Briefly, A549 cells were treated for 1 h with 50 μg/ml of CHX, and then inoculated with rgRSV grown from *METTL3*-KD U2OS or wt U2OS cells at an MOI of 0.5. As shown in **Fig 4B**, the m^6^A-deficient rgRSV grown in *METTL3*-KD U2OS cells did induce significantly more IFN-β mRNA in the absence of protein synthesis and virus replication, than m^6^A-sufficient rgRSV grown in wt U2OS cells (*P*<0.05).

### m^6^A-deficient rgRSV mutants are attenuated in replication in vitro

**Fig 5:**
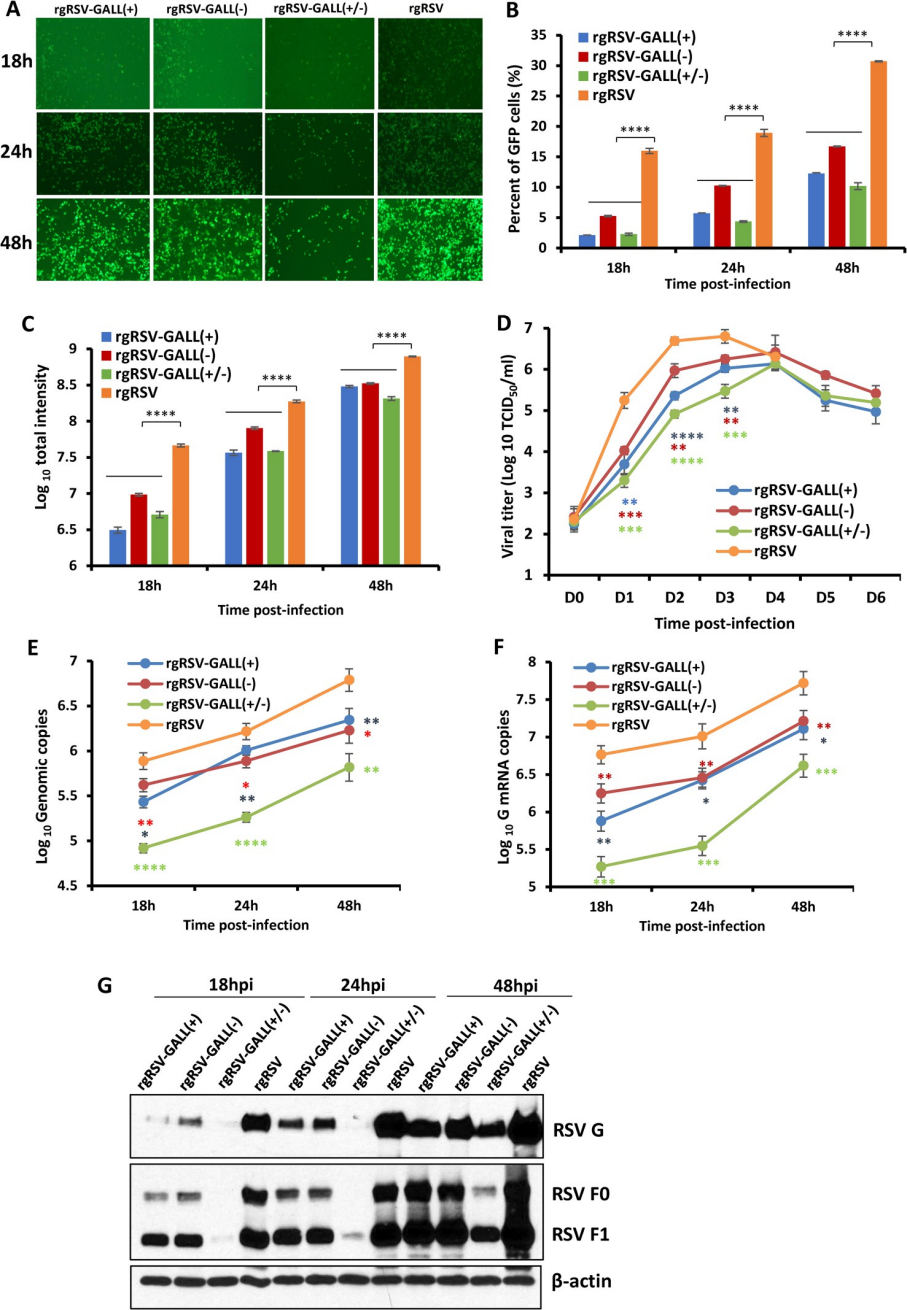
Replication of m^6^A-deficient RSVs is attenuated in A549 cells. (A) Delayed GFP expression by rgRSV mutants in A549 cells. Confluent A549 cells were infected by each rgRSV at an MOI of 0.1, and GFP expression was monitored at the indicated times by fluorescence microscopy. (B-C) Quantification of GFP positive cells by flow cytometry. Confluent A549 cells were infected by each rgRSV (MOI of 0.1). At the indicated time points, cells were trypsinized and GFP positive cells (B) and GFP intensity (C) were quantified by flow cytometry. (D) Single-step growth curve of rgRSV mutants. A549 cells in 24-well-plates were infected with each recombinant rgRSV at an MOI of 0.1. The viral titer was determined by TCID_50_ assay in HEp-2 cells. (E) RSV genome RNA replication. At 18, 24, and 48 h post-infection, total RNA was purified from rgRSV-infected cells using TRizol, and genome RNA was quantified by real-time RT-PCR using specific primers annealing to the RSV leader sequence and GFP gene. (F) RSV G mRNA transcription. Viral mRNA was quantified by real-time PCR using primers annealing to the G. (G) Western blot analysis of RSV F and G protein expression. A549 cells were infected with the parental rgRSV or rgRSV mutants at an MOI of 0.1. At 18, 24, and 48 h post-inoculation, total cell lysates were harvested and subjected to Western blotting using a monoclonal antibody against RSV F or G protein. The RNA copy and viral titers are the geometric mean titer (GMT) of three independent experiments ± standard deviation. Western blots shown are the representatives of three independent experiments. Data were analyzed using Student’s *t*-test and *P < 0.05; **P < 0.01; ***P < 0.001; ****P < 0.0001.

Since viral m^6^A methylation positively regulated RSV replication and gene expression [37], rgRSV mutants lacking m^6^A methylation would likely be attenuated in replication. To test this, A549 cells were infected with rgRSV, rgRSV-GALL(+), rgRSV-GALL(-) or rgRSV-GALL(+/-) at an MOI of 0.1, and viral replication, gene expression, and RNA synthesis were analyzed. All rgRSV mutants expressed significantly less GFP compared to the parental rgRSV **(Fig 5A**). The combined mutant rgRSV-GALL(+/-) was the most defective virus for GFP expression (**Fig 5A**). The number of GFP positive cells and the intensity of GFP were monitored by flow cytometry. All rgRSV mutants produced fewer GFP positive cells (**Fig 5B)** and infected cells were significantly less bright (**Fig 5C**) compared to the parental rgRSV. A single-step growth curve showed that all rgRSV mutants had delayed replication kinetics and had approximately 0.5 log reductions in peak viral titer compared to the rgRSV (**Fig 5D**).

We also examined viral RNA synthesis in virus-infected cells by real-time RT-PCR. All rgRSV mutants had significant reductions (1.0–1.5 log RNA copies) in genome replication (**Fig 5E**) and G mRNA synthesis (**Fig 5F**). We also examined viral protein expression in virus-infected cells. A549 cells were infected with each virus at an MOI of 0.1. Cells were lysed at 18, 24, and 48 h and viral protein expression was analyzed by Western blot using antibody against G and F proteins. All rgRSV mutants had defects in G and F protein synthesis, particularly at the early time points **(Fig 5G**). Together, all rgRSV mutants were significantly attenuated in A549 cells and rgRSV-GALL(+/-) was the most attenuated virus.

**Fig 6:**
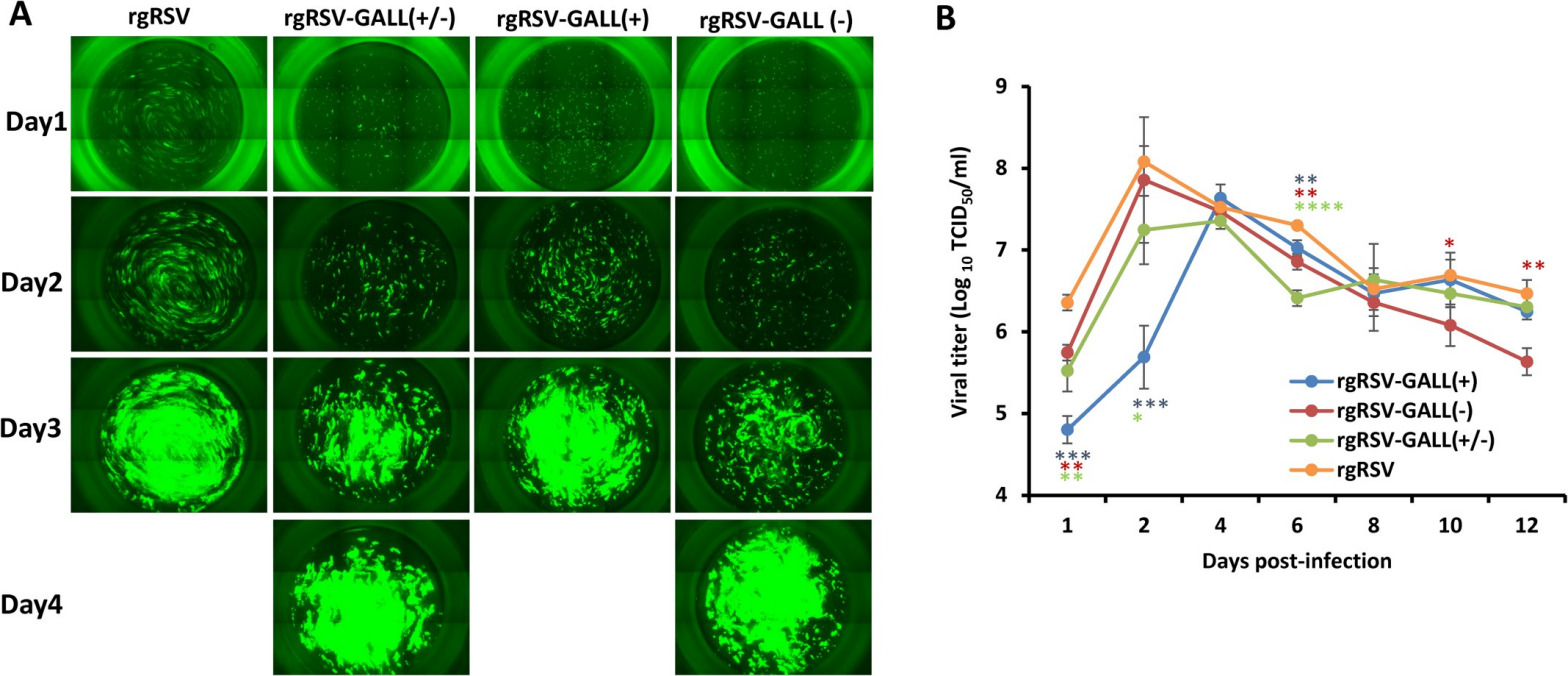
RSV replication in HBE cultures. (A) Spreading of m^6^A-deficient rgRSVs in HBE culture. HBE cultures were infected by 400 TCID_50_ of each rgRSV mutant. At the indicated time, virus spread was monitored by fluorescence microscopy. Representative micrographs at each time point are shown. (B) Virus release from m^6^A-deficient rgRSV-infected HBE culture. HBE cultures were infected by 400 TCID_50_ of each rgRSV. After virus inoculation, supernatants were collected on day 1 and every 2 days until day 12 post-inoculation. Infectious virus in supernatants was determined by TCID_50_ assay. Viral titers are the geometric mean titer (GMT) of three independent experiments ± standard deviation. Data were analyzed using Student’s *t*-test and *P < 0.05; **P < 0.01; ***P < 0.001; ****P < 0.0001.

We also tested replication of rgRSVs in primary well-differentiated human bronchial epithelial (HBE) cultures, the ex vivo model for RSV infection [37,53]. Briefly, HBE cultures were infected with 400 TCID_50_ of each virus, GFP expression was monitored daily and the released virus was measured by TCID_50_ assay until day 4 after infection. All rgRSV mutants were defective in spreading and GFP expression at days 1 and 2 but gradually reached saturation at day 4 post-inoculation (**Fig 6A**). Again, rgRSV-GALL(+/-) was the most defective virus in spreading in HBE culture. In addition, all rgRSV mutants had a delay in virus release in HBE cultures compared to rgRSV (**Fig 6B**).

### Restoration of the replication of m^6^A-deficient rgRSVs in RIG-I and MAVS-knockout A549 cells

**Fig 7:**
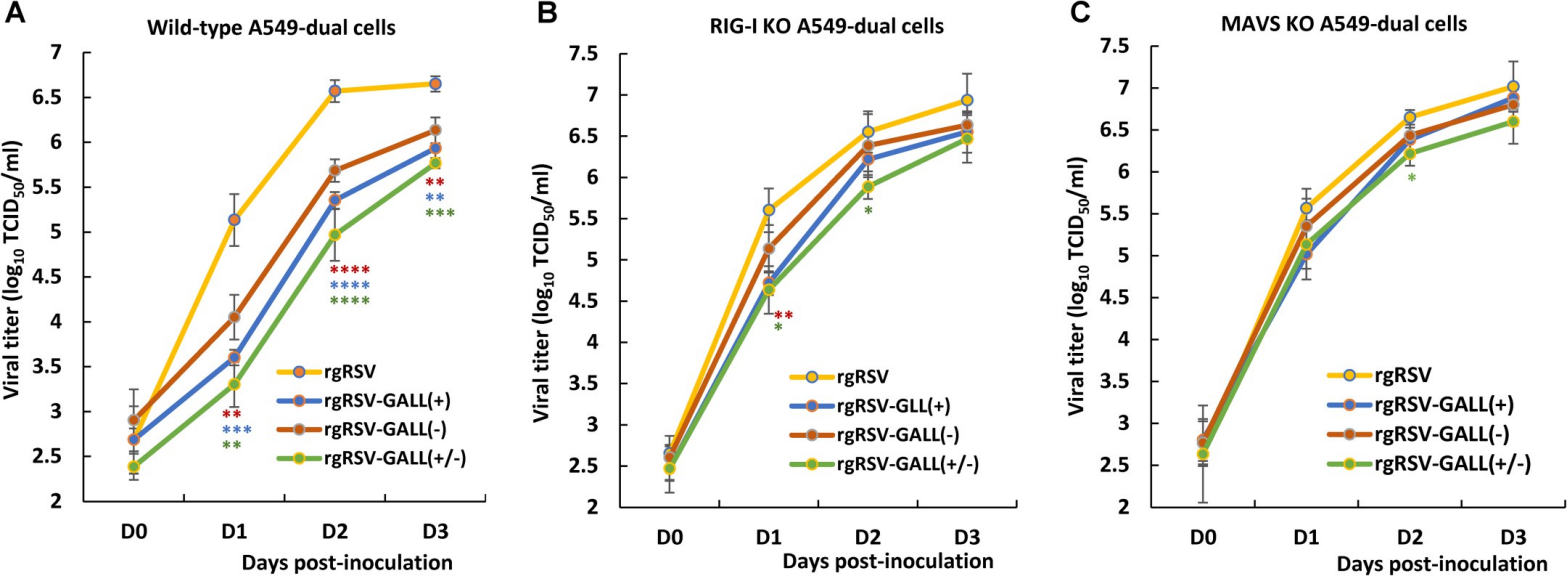
Restoration of the replication of m^6^A-deficient rgRSVs in RIG-I and MAVS-knockout A549 cells. Replication kinetics of m^6^A-deficient rgRSVs in WT (**A**) RIG-I-knockout (**B**) or MAVS-knockout (**C**) A549 cells. Cells in 24-well plates were infected by each rgRSV at an MOI of 1.0, virus was harvested at day 0–3 post-infection, and viral titer was determined by TCID_50_ assay in HEp-2 cells. Viral titers are the geometric mean titer (GMT) of three independent experiments ± standard deviation. Viral titer of each rgRSV at each time point was compared with that of the parental rgRSV. Statistical significance was determined by two-sided Student’s *t*-test. *P<0.5, **P < 0.01, ***P < 0.001, ****P < 0.0001.

We next compared the replication kinetics of rgRSV mutants and rgRSV in wild-type A549-Dual cells, RIG-I KO A549-Dual cells, and MAVS KO A549-Dual cells. Briefly, each cell line was infected by each virus at an MOI of 0.1, and viral titer at days 0–3 was determined by TCID_50_ assay. All three rgRSV mutants showed significant growth defects in WT A549-Dual cells (**Fig 7A**). However, the growth of three rgRSV mutants was restored in RIG-I-KO (**Fig 7B**) and MAVS-KO (**Fig 7C**) A549-Dual cells. Therefore, m^6^A-deficient rgRSVs is attenuated in replication in wt A549 cells but their replication is restored in A549 cells lacking IFN response. These results further support the notion that RIG-I is involved in recognition of m^6^A-deficient RSV RNA.

### m^6^A-deficient rgRSV mutants induce significantly higher innate immunity in vivo

**Fig 8:**
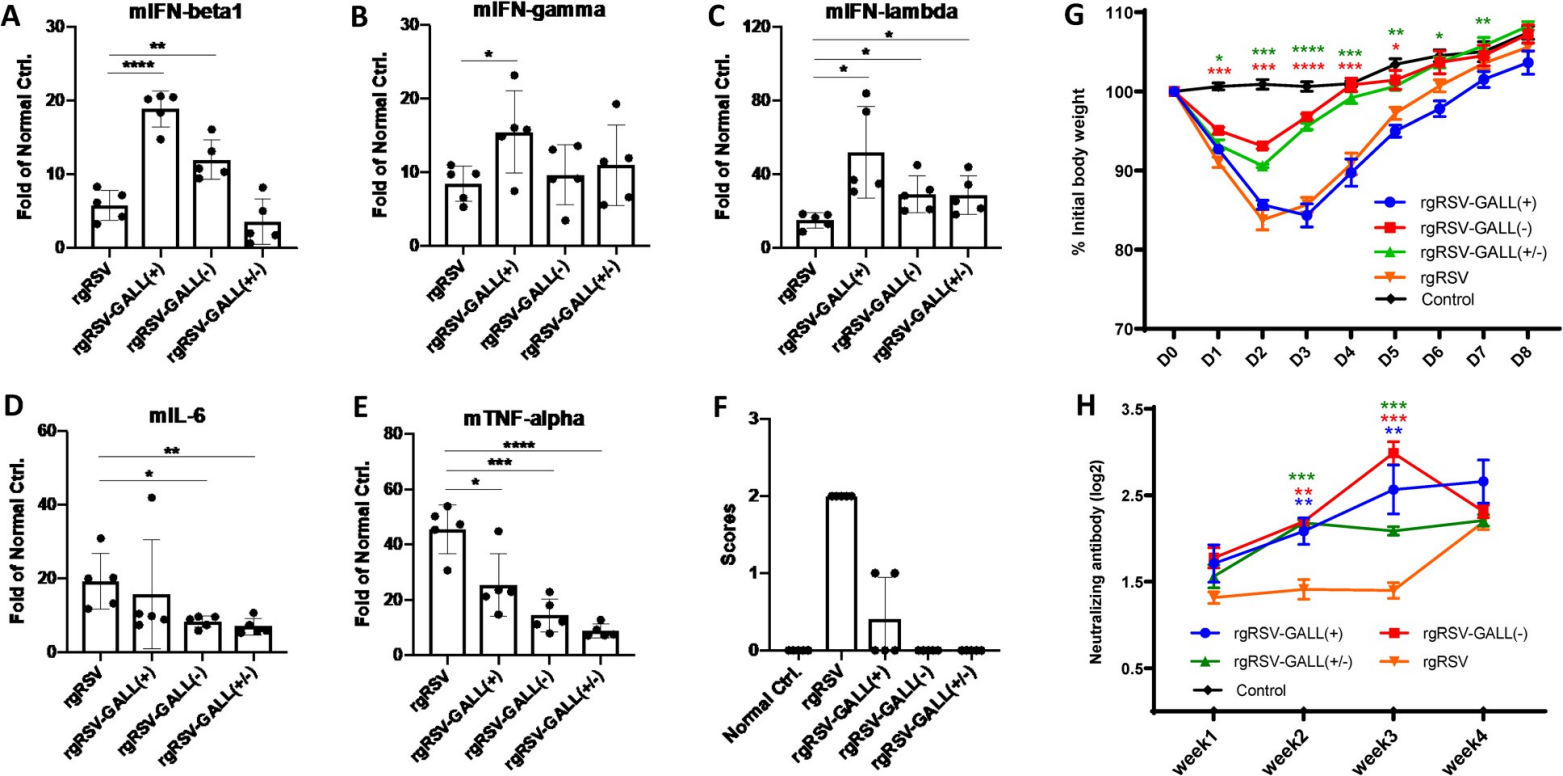
Innate immune response and antibody response of m^6^A-deficient rgRSVs in mice. (A-E) Innate immune response of m^6^A-deficient rgRSVs in mice. 4-week old BALB/c mice were intranasally inoculated with PBS or PBS containing 5×10^5^ TCID_50_ of rgRSV, rgRSV-GALL(+), rgRSV-GALL(-), or rgRSV-GALL(+/-). At 48h post-inoculation, all mice were euthanized, mouse IFNβ (mIFNβ) (A), mIFN-γ (B), mIFN-λ (C), mIL-6 (D), and mTNF-α (E) were quantified by real-time RT-PCR. (F) Clinical signs of mice inoculated with recombinant viruses. The severity of clinical signs were scored. Grade 3 (severe) was characterized by ruffled fur, hyperexcitability, tremors, and circling; grade 2 (moderate) was characterized by ruffled fur, hunched posture, and reduced food and water intake; grade 1 (mild) was characterized by mild ruffled fur; and grade 0 was defined as no symptoms. (G) Dynamics of mouse body weight after inoculation with m^6^A-deficient rgRSV. Five 4-6-week-old female BALB/c mice in each group were intranasally inoculated with PBS or 10^6^ TCID_50_ of rgRSV-GALL(+), rgRSV-GALL(-), rgRSV-GALL(+/-), and parental rgRSV. The body weight for each mouse was evaluated at indicated time points. The average body weights of five mice are shown. (H), The RSV-neutralizing antibody titer was determined using a plaque reduction assay. Cytokine data are the fold change of mice inoculated rgRSV or each rgRSV mutant relative to the normal control mice inoculated with PBS. Data are the mean of 5 mice (*n* = 5) ± standard deviation. Antibody titers are the geometric mean titer (GMT) of 5 mice (*n* = 5) ± standard deviation. *P<0.5, **P < 0.01, ***P < 0.001, ****P < 0.0001. Data were analyzed using Student’s *t*-test.

To determine whether m^6^A-deficient rgRSV mutants induce higher innate immunity in vivo, BALB/c mice were inoculated intranasally with each m^6^A-deficient rgRSV and the parental rgRSV at a dose of 5×10^5^ TCID_50_ per mouse. At day 2 post-inoculation, mice were euthanized, and IFN-β (type I), IFN-γ (type II), IFN-λ (type III), IL-6, and TNF-α in lung tissue were quantified by real-time RT-PCR. We found that IFN-β (**Fig 8A**) was significantly increased in rgRSV-GALL (+) and rgRSV-GALL (-), IFN-γ (**Fig 8B**) was significantly increased in rgRSV-GALL(+), and IFN-λ (**Fig 8C**) was significantly increased in all three rgRSV mutants compared to the parental rgRSV. Interestingly, IL-6 (**Fig 8D**) and TNFα (**Fig 8E**), the signature cytokines for lung inflammation, were significantly reduced for all three rgRSV mutants compared to rgRSV [except IL-6 for rgRSV-GALL(+)]. These results indicate that rgRSV mutants stimulated significantly higher IFN while reducing inflammatory cytokine responses in vivo.

### m^6^A-deficient rgRSV mutants trigger stronger neutralizing antibody and T cell responses

Since m^6^A-deficient rgRSV mutants induced higher type I IFN, we hypothesize that these mutants will modulate adaptive immunity. We chose mice for this experiment because of the availability of immune reagents. Briefly, mice were intranasally inoculated with 1.0×10^6^ TCID_50_ of rgRSV-GALL(+), rgRSV-GALL(-), rgRSV-GALL(+/-), parental rgRSV, and PBS, respectively. Consistent with previous observations [54], mice inoculated with rgRSV showed significant clinical signs (such as ruffled fur and a hunched posture, average score of 2) (**Fig 8F**) and caused significant body weight loss (**Fig 8G**). Interestingly, mice in rgRSV-GALL(+) groups showed no or mild clinical signs (average score of 0.4) (**Fig 8F**) even though they had a similar level of body weight loss as the rgRSV group (**Fig 8G**). However, body weight in both groups had recovered by day 8. Mice in the rgRSV-GALL(-) and rgRSV-GALL(+/-) groups did not exhibit any clinical signs (score of 0) (**Fig 8F**) and had significantly less body weight loss compared to the rgRSV group (*P*<0.05) and had recovered by day 4 (**Fig 8G**). These results demonstrate that m^6^A-deficient rgRSVs are attenuated to various degrees in mice compared to rgRSV.

After virus inoculation, serum samples were collected weekly from each mouse and neutralizing antibodies were measured (**Fig 8H**). Notably, all three m^6^A-deficient rgRSV groups showed significantly higher neutralizing antibodies at weeks 2 and 3 after virus inoculation. This result suggests that m^6^A-deficient rgRSV mutants induce higher neutralizing antibodies more quickly in mice compared to rgRSV.

**Fig 9:**
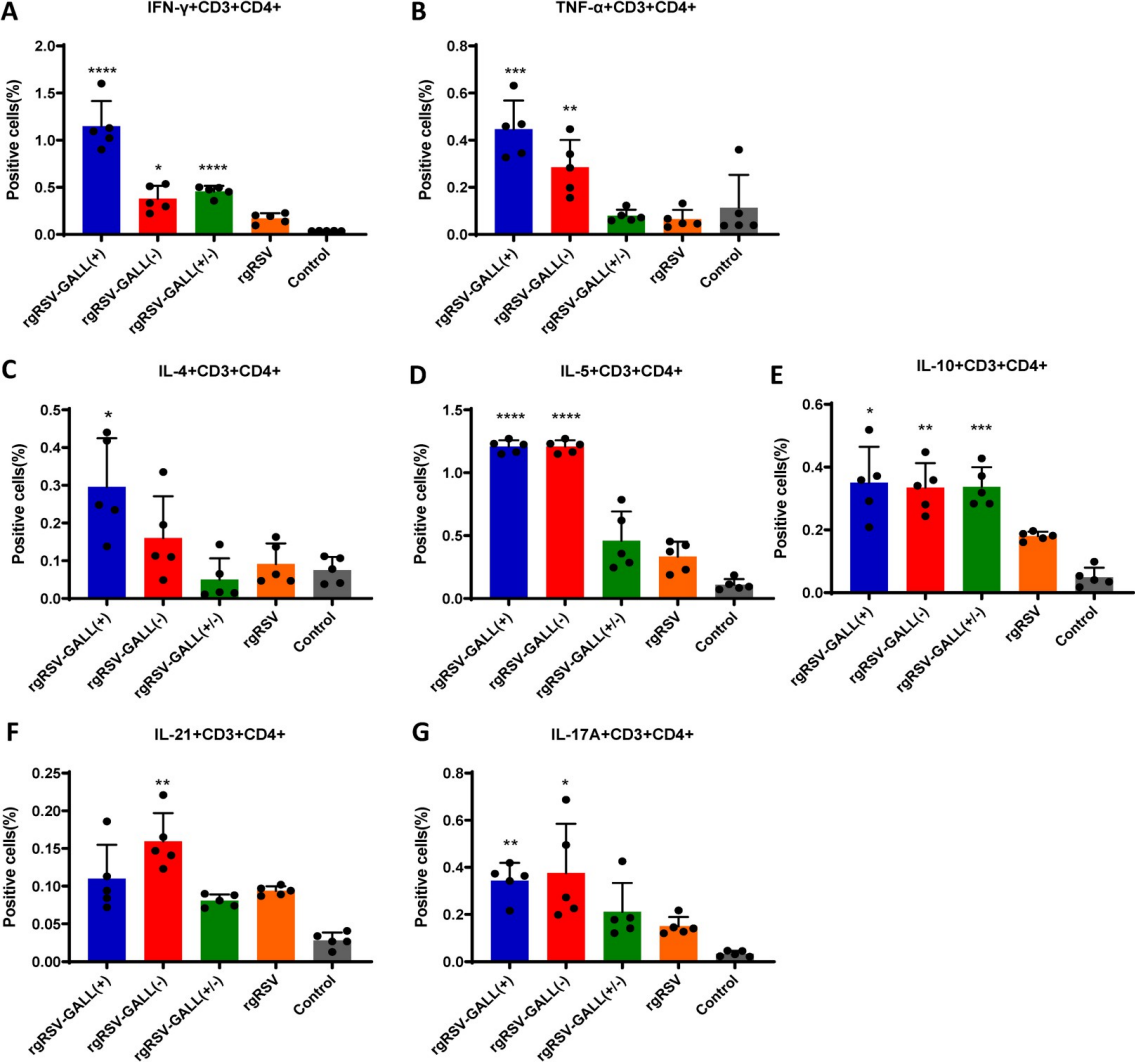
Viral m^6^A methylation modulates RSV-specific T cell response. Five-week-old BALB/c mice were immunized with each rgRSV mutant (5 mice per group). Mice were euthanized at 4 weeks post-immunization, the spleen was isolated from each mouse, homogenized, a cell suspension prepared, split into three wells (triplicate per mouse) and cultured in 96-well microtiter plates in the presence of 20 μg/ml of RSV pre-fusion protein for 5 days. The frequencies of RSV-specific Th1 (IFN-γ+CD4+ and TNF-α+CD4+) (A-B), Th2 cells (IL-4+CD4+, IL-5+CD4+, IL-10+CD4+) (C-E), Tfh (IL-21+ CD4+) (F) and Th17 (IL-17A+ CD4+) (G) cells were determined by flow cytometry after intracellular staining with the corresponding anti-cytokine. Error bars represent SD from *n*  =  5 mice. *P<0.5, **P < 0.01, ***P < 0.001, ****P < 0.0001. Data were analyzed using Student’s *t*-test.

At week 4 post-inoculation, mice were terminated and spleen cells were isolated to assess T cell activation. Th1 cells play crucial roles in preventing virus infections by producing cytokines that enhance the generation of complement fixing antibodies and cytotoxic T cells. The analysis of RSV F protein-specific CD3+CD4+ cells producing Th1 cytokines by flow cytometry (**Fig 9A** and **9B**) demonstrated that mice immunized with all three m^6^A-deficient rgRSVs produced significantly more IFN-γ T cells (CD4+IFN-γ+) than mice immunized with the parental rgRSV. Interestingly, TNF-α producing T cells (CD4+TNF-α+) were detected in the spleens of mice immunized with rgRSV-GALL(+) and rgRSV-GALL(-), but not with rgRSV-GALL(+/-) or rgRSV.

Th2 cells trigger cytokines that are involved in the production of antibodies against extracellular pathogens. Follicular T helper cells (Tfh) and Th17 cells are important in promoting affinity maturation of antibodies, and the signature cytokines are interleukin 21 and IL-17A, respectively. As shown in **Fig 9C–9E**, rgRSV-GALL(+) triggered slightly more CD4+IL-4+ cells than the other viruses. Both rgRSV-GALL(+) and rgRSV-GALL(-) generated significantly more CD4+IL-5+ and CD4+IL-10+ cells compared to the parental rgRSV. All rgRSV mutants produced much stronger CD4+IL-10+ than rgRSV. Additionally, slightly more Tfh cells were observed in the rgRSV-GALL(-) group whereas significantly higher Th17 responses (CD4+IL-17A+) were observed after immunized with rgRSV-GALL(+) and rgRSV-GALL(-) (**Fig 9F** and **9G**). These results demonstrate that m^6^A-deficient rgRSVs enhanced most types of T cell responses better than the parental rgRSV.

### m^6^A-deficient rgRSV mutants are attenuated in cotton rats and provide full protection against RSV challenge

**Fig 10:**
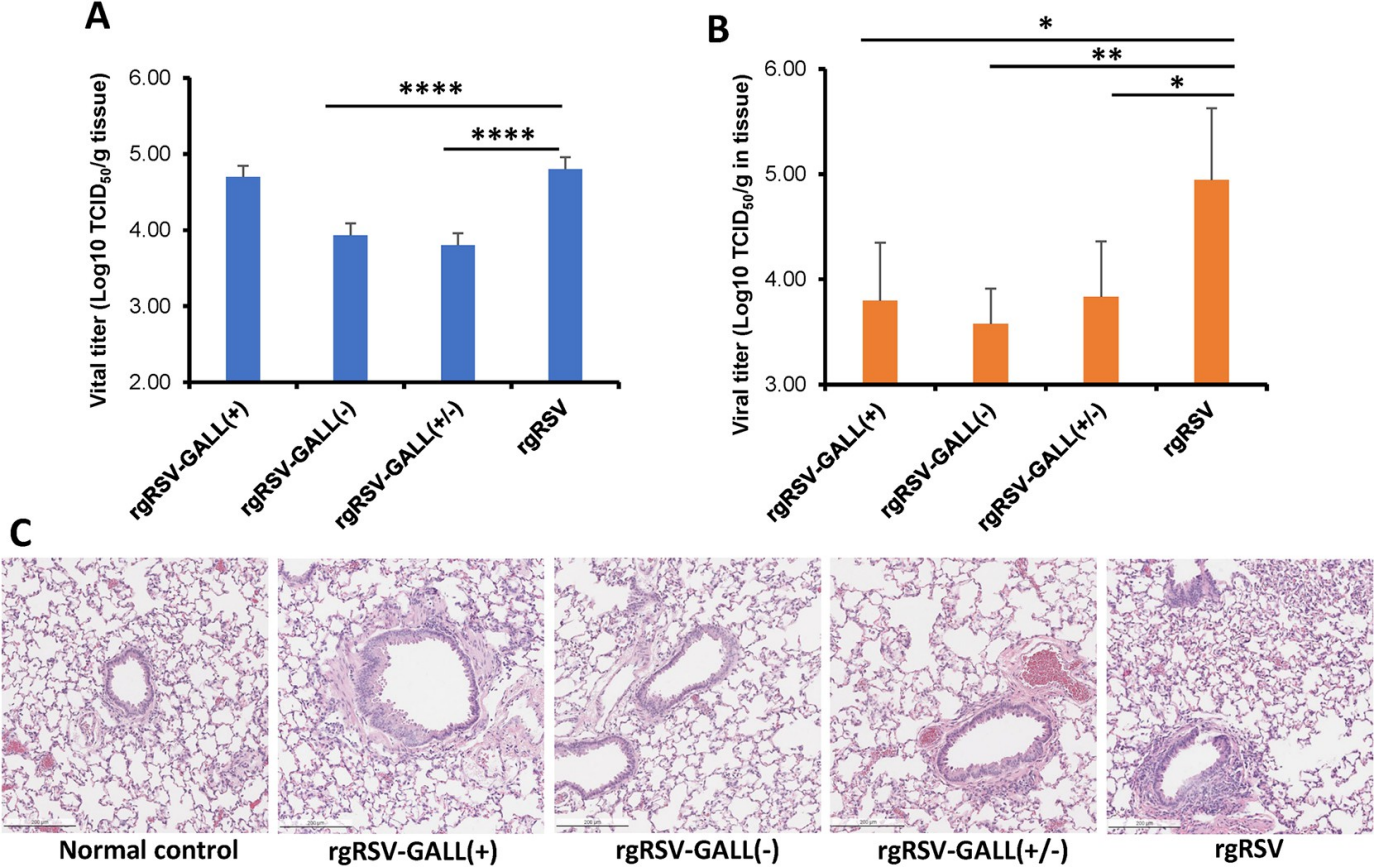
Replication of m^6^A-deficient rgRSVs in cotton rats. (A-B), Replication of rgRSV mutants in cotton rats. Four-week-old SPF cotton rats were inoculated intranasally with 2.0×10^5^ TCID_50_ of each rgRSV. At day 4 post-infection, the cotton rats were sacrificed, and lungs (A) and nasal turbinate (B) were collected for virus titration by TCID_50_ assay. (C), Lung histology from RSV-infected cotton rats. Hematoxylin-eosin (HE) staining of lung tissue was shown. Histological images were taken under light microscopy. Micrographs with ×20 magnification (scale bar of 500 μm) are shown.

We next determined the replication of rgRSV mutants in cotton rats, as RSV replicates more robustly in the lungs and nasal turbinate of cotton rats than those of mice [55]. Briefly, cotton rats were intranasally inoculated with 2×10^5^ TCID_50_ of each rgRSV, cotton rats were terminated at day 4, and viral titer in lung and nasal turbinate were determined. Recombinant rgRSV-GALL(+) had no significant reduction in lungs whereas rgRSV-GALL(-) and rgRSV-GALL(+/-) had a 1 log reduction in lungs compared to rgRSV (**Fig 10A**). All three m^6^A-deficient rgRSV had a 1.1–1.4 log virus reduction in nasal turbinates (**Fig 10B**). The results suggest that replication of rgRSV-GALL(+) is more attenuated in the nose than in the lungs whereas rgRSV-GALL(-) and rgRSV-GALL(+/-) have similar levels of attenuation in replication in both lung and nose. Histopathologic examination showed that lungs from rgRSV-infected cotton rats had mild to moderate pathological changes characterized by few leukocytes surrounding bronchioles and blood vessels, peribronchial lymphoplasmocytic infiltrates, and evidence of occasional bronchiolar goblet cell hyperplasia with granulocyte exocytosis (**Fig 10C)**. All lungs from rgRSV mutant-infected groups had mild pathological changes (**Fig 10C**). Therefore, these rgRSV mutants had various degrees of attenuation in replication in cotton rats, however, rgRSV-GALL(+/-) was the most attenuated mutant.

**Fig 11:**
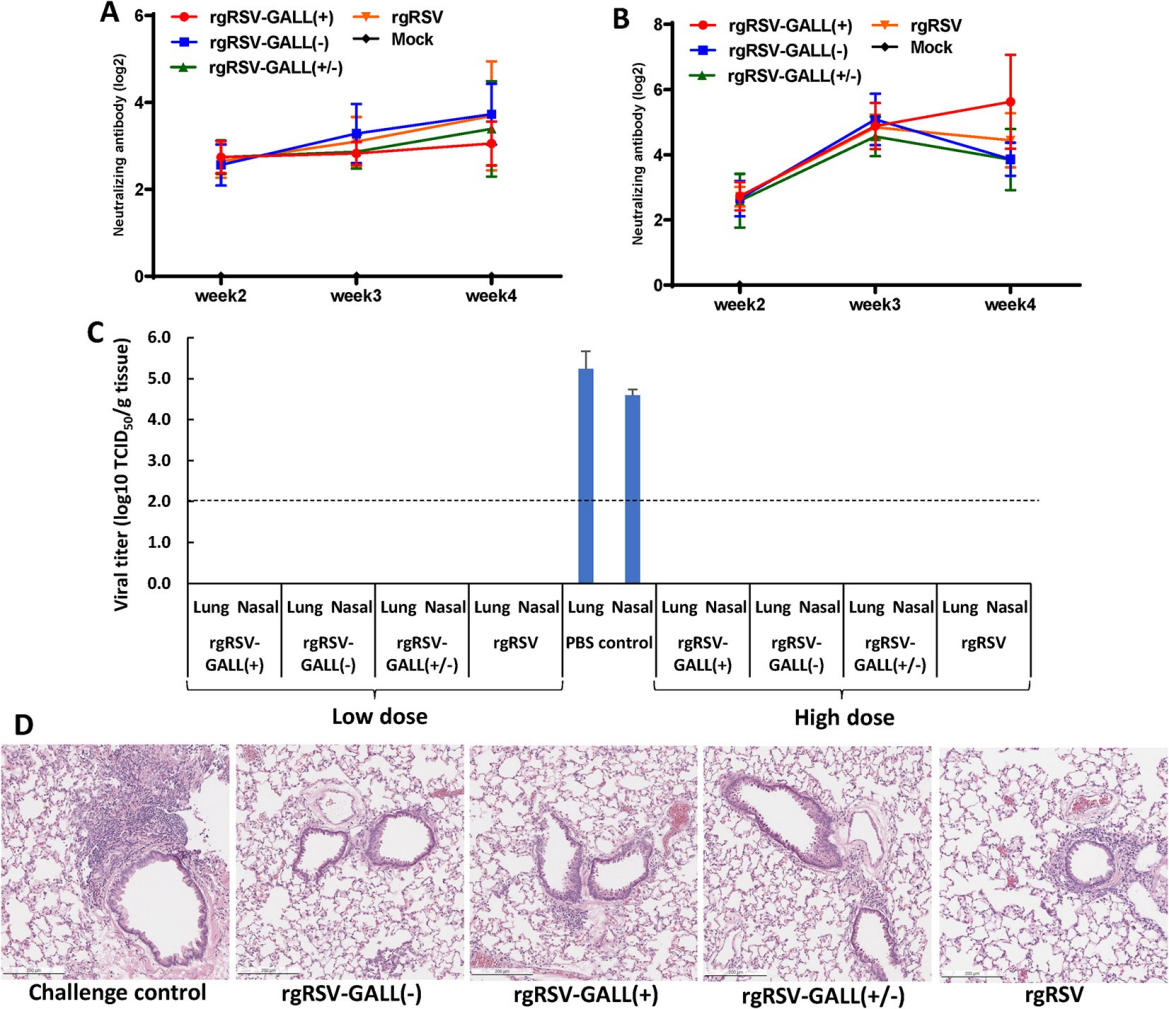
Immunogenicity of m^6^A-deficient rgRSVs in cotton rats. Four-week-old SPF cotton rats were inoculated intranasally with 2.0×10^3^ or 2.0×10^5^ TCID_50_ of each rgRSV. Blood samples were collected from each rat weekly by retro-orbital bleeding. The RSV-neutralizing antibody titer was determined using a plaque reduction assay (A and B). At week 4 post-immunization, cotton rats were challenged with 2.0×10^5^ TCID_50_ rgRSV. At day 4 post-challenge, the cotton rats were sacrificed, and lungs and nasal turbinate were collected for virus titration by TCID_50_ assay (C). Antibody titers and viral titers are the geometric mean titer (GMT) of 5 animals ± standard deviation. The detection limit for virus in tissue is 2.0 log TCID_50_/g tissue. Error bars represent SD from *n*  =  5 mice. *P<0.5, **P < 0.01, ***P < 0.001, ****P < 0.0001. (D), Lung histology from RSV-infected cotton rats. Hematoxylin-eosin (HE) staining of lung tissue was shown. Histological images were taken under light microscopy. Micrographs with ×20 magnification (scale bar of 500 μm) are shown.

We also determined the immunogenicity of each rgRSV mutant in cotton rats. Briefly, cotton rats were intranasally immunized with 2×10^3^ TCID_50_ or 2×10^5^ TCID_50_ of each virus. Blood was collected from each cotton rat weekly for quantification of neutralizing antibody. All three m^6^A-deficient rgRSVs generated similar levels of neutralizing antibodies compared to the parental rgRSV at both low (**Fig 11A**) and high doses (**Fig 11B).** At week 4 post-immunization, animals were challenged with 2×10^5^ TCID_50_ of rgRSV and cotton rats were euthanized at day 4 for analysis of viral replication in lungs and nasal turbinates. The unimmunized but challenged groups had 4–5 log TCID_50_ viral titer in lungs and nasal turbinate. All immunized animals were completely protected from rgRSV challenge. No infectious RSV was detected in lungs or nasal turbinates from animals immunized with low or high dose (**Fig 11C**). Lung tissues from each group were examined histologically. Lungs from the unimmunized challenged control had mild to moderate pathological changes whereas lungs from all immunized challenged groups were comparably mild (**Fig 11D**). No enhanced lung damage was observed in immunized animals. These data demonstrate that all three m^6^A-deficient rgRSVs are highly immunogenic in in cotton rats.

## Discussion

A live attenuated vaccine is one of the most promising vaccine candidates for RSV. However, most live attenuated RSV vaccine candidates are less immunogenic than the wild type virus [12,56]. Furthermore, a well-known challenge with RSV is that even natural infection does not induce long-lived immunity [12,57]. Therefore, enhanced immunogenicity would likely be necessary for a successful live-attenuated RSV vaccine. RSV NS1 and NS2 proteins are known to strongly inhibit innate immunity, which in turn dampens the adaptive immune responses [19,58]. Thus, some of the current efforts to improve the innate immune response to RSV are focused on these two nonstructural proteins. Recombinant RSVs with deletion of the entire NS1 and/or NS2 protein or mutations in NS1 do induce a higher innate immune response [19,20,22]. However, this approach impairs the ability of the virus to grow in cell culture, even in Vero cells which lack an interferon response [20,22]. No viruses with modifications in NS1 or NS2 proteins that improve immunogenicity while maintaining robust replication in Vero cells have been described.

In this study, we found that m^6^A methylation of viral RNA modulates both innate and adaptive immune responses to RSV. Specifically, we found that m^6^A-deficient virion RNA and m^6^A-deficient virus infection induce significantly higher IFN responses in vitro and in vivo and that m^6^A-deficient rgRSVs induced higher neutralizing antibody and T cell immune responses in mice, providing complete protection against RSV challenge in cotton rats. A model consistent with our findings is depicted in **S6 Fig**. These results support the hypothesis that the higher innate immune response induced by RSV, in turn, induces a stronger adaptive immune response. Thus, inhibition of viral m^6^A methylation is a novel approach to enhance both the innate and adaptive immune responses of RSV vaccine candidates.

Viral m^6^A methylation is an emerging field. Recent studies have shown that viral m^6^A methylation can play an anti-viral or a pro-viral role via poorly understood mechanism(s) [59]. In some viruses, m^6^A methylation in viral mRNA enhances its stability and therefore protein translation, enhancing viral replication [60]. In other viruses, viral m^6^A methylation appears to promote viral mRNA decay or inhibit virion packaging thereby inhibiting viral replication [35,61]. As a non-segmented negative-sense (NNS) RNA virus, the RSV genome and antigenome (replicative intermediate) are not capped or polyadenylated and cannot be directly translated into viral protein. Unlike the role of m^6^A in viral mRNA, we showed that m^6^A methylation in the RSV genome and antigenome strongly modulates innate immunity. We proved this concept using two strategies. First, by growing RSV in *METTL3*-knockdown cells, we generated RSV virion RNA that was defective in m^6^A methylation without altering the nucleotide sequence of the viral RNA. As a second approach, using synonymous mutations, we generated m^6^A-deficient RSVs lacking m^6^A-sites in the antigenome, genome, or both. In all cases, m^6^A-deficient RSV virion RNA transfection and virus infection induced significantly higher type I IFN responses compared to wt RSV. Mechanistically, these m^6^A-deficient RSV RNA directly stimulate RIG-I mediated IFN signaling pathway such as the enhancement of RIG-I expression, RIG-I binding affinity, RIG-I ubiquitination, and IRF3 phosphorylation. Our results demonstrate that RSV genome and antigenome lacking m^6^A methylation are recognized by host innate immune mechanisms as non-self RNA, which trigger a higher IFN response. These phenotypes in RSV are similar to our previous observations of m^6^A-deficient hMPVs [38]. Thus, the role of virion RNA m^6^A methylation in innate immunity is conserved in these two important pneumoviruses. Our results, coupled with the recent observations that m^6^A modified circular RNA [62]and m^6^A modified HBV, HCV, and HIV RNA avoid RIG-I signaling [63,64], suggest that m^6^A methylation of RNA is a molecular marker for host innate immunity to discriminating self from non-self RNA through the RNA sensor RIG-I for many different viruses.

We also found that m^6^A-deficient RSV mutants induce stronger IFN responses in mice. All three m^6^A-deficient RSV mutants induce a significantly higher IFN-λ response, RSV-GALL(+) and rgRSV-GALL(+/-) induce significantly higher IFN-β, and RSV-GALL(+) has a significantly higher IFN-γ response compared to rgRSV. IL-6 and TNFα are the signature cytokines of lung inflammation and have been associated with severe RSV diseases such as severe bronchiolitis and encephalopathy [65,66]. Interestingly, these m^6^A-deficient RSV mutants had reduced levels of IL-6 and TNFα in lungs, thereby increasing their safety profile as vaccines.

A key finding of this study is that viral m^6^A also modulates adaptive immunity. We found that all three m^6^A-deficient RSV mutants are more immunogenic than the parental rgRSV in mice. All three m^6^A-deficient RSV mutants induced significantly higher neutralizing antibodies at weeks 2 and 3 post-inoculation compared to the parental rgRSV. All three m^6^A-deficient RSV mutants induced increased numbers of Th1 cells, which produce interferon gamma (IFN-γ), an essential cytokine in the viral cell-mediated immune response [67], and enhanced the Th2 cell response, which selectively produces IL-4, IL-5, and IL-10 that participate in the development of humoral immunity [67]. In addition, m^6^A-deficient RSVs also induced a higher level of Tfh and Th17 cells, which are important in promoting affinity maturation of antibodies.

In the 1960s, the formalin-inactivated RSV (FI-RSV) vaccine resulted in an enhanced lung damage (ERD) in human trials [4,5]. A predominant Th2 response was found in these cases [4,5]. Thus, it is thought that the imbalance between Th1 and Th2 cells may have contributed to the ERD [68,69]. Since both Th1 and Th2 responses were enhanced for m^6^A-deficient RSV mutants, the ultimate balance between Th1 and Th2 cells was not significantly altered. In addition, the three m^6^A-deficient RSV mutants demonstrated a degree of attenuation in mice and cotton rats, consistent with the fact that viral m^6^A methylation positively regulates RSV replication and gene expression [46]. We chose cotton rats for immunization and challenge experiments because they are highly permissive for RSV infection [70]. Cotton rats immunized with low or high doses of these m^6^A-deficient RSV mutants were completely protected from RSV challenge. Perhaps, cotton rats are unable to differentiate the immunogenicity of these m^6^A-deficient RSV mutants and parental rgRSV even though the immunization dose has been reduced to as low as 2.0×10^3^ TCID_50_ per cotton rat. Nevertheless, these results demonstrate that m^6^A-deficient RSV mutants retain high immunogenicity despite the fact that they are significantly attenuated in replication in nose and lung of cotton rats. Histologic examination of lung tissues showed that no ERD was observed in cotton rats challenged with RSV, suggesting that m^6^A modulates adaptive immunity without inducing harmful immune responses and lung pathology.

Based on the balance between the safety, innate immunity, and immunogenicity, rgRSV-GALL(-) appears to be the best at inducing a type I IFN response, followed by rgRSV-GALL(+) and rgRSV-GALL(+/-), as a backbone for a live attenuated vaccine. It should be noted that m^6^A-deficient RSV mutants may not be attenuated enough for live attenuated vaccine candidates. One future direction is to combine the m^6^A mutations with other known attenuating mutations (such as viral mRNA cap methylation mutations or cold-adapted mutations) to generate an RSV vaccine candidate that has an optimal balance between attenuation and immunogenicity.

Recent studies have shown that *IFNβ* mRNA contains m^6^A methylation and that the m^6^A level of IFNβ is reduced following repression of METTL3, stabilizing the *IFNβ* mRNA and leading to higher levels of IFN production upon infection by human cytomegalovirus (HCMV) [71,72]. Interestingly, our m^6^A-seq data showed that *IFNβ* mRNA contains m^6^A methylation in A549 cells but not HeLa cells (GEO: GSE125803) [46], suggesting that m^6^A modification patterns may be cell-type specific. In addition, we found that other IFN-related genes including RIG-I, MDA5, and IRF3 are m^6^A modified in both RSV-infected A549 cells and HeLa cells (GEO: GSE125803) [46]. Further studies are needed to understand the roles of m^6^A modifications of these IFN-related genes in the IFN response. Another study showed that loss of *METTL3* and m^6^A methylation in hematopoietic stem cells activated an aberrant innate immune response, mediated by the formation of endogenous double-stranded RNAs (dsRNAs) [73]. However, our results suggest that there may be other possibilities. For example, replication of RSV in *METTL3*-KD cells will lead to the generation of m^6^A-deficient genome and antigenome, which are recognized as non-self RNA, thereby stimulating higher type I IFN production. Although we only observed 24–27% reductions in m^6^A content in RSV virion RNA in both *METTL3*-KD U2OS and A549 cells, transfection of these m^6^A-deficient RNA into cells results in a significant increase in type I IFN signaling compared to wt virion RNA. It is possible that METTL3 KD may affect the key m^6^A sites in RSV RNA. Interestingly, we recently found that several NNS RNA viruses including Sendai virus (SeV), measles virus (MeV), vesicular stomatitis virus (VSV), and human metapneumovirus (hMPV) had different degrees of reduction in viral m^6^A methylation in *METTL3*-KD U2OS cells [74]. Specifically, SeV, MeV, hMPV, and VSV grown in *METTL3*-KD U2OS cells led to a reduction of 80%, 80%, 60%, and 20% in viral RNA m^6^A content, respectively. The degree of m^6^A reduction was correlated with the level of IFN induction [74]. Consistent with our results, it was shown that knockdown of the m^6^A writer enzymes (METTL3 and METTL14) leads to an increase in hepatitis B virus and hepatitis C virus RNA recognition by RIG-I [63]. Similarly, m^6^A eraser proteins (FTO and ALKBH5) treated HIV-1 RNA induces a significantly higher type I interferon response in differentiated human monocytic cells and primary monocyte-derived macrophages [64]. Finally, it was recently showed that depletion of METTL3 decreases m^6^A levels in SARS-CoV-2 RNA and host genes and that m^6^A reduction in SARS-CoV-2 RNA increases RIG-I binding and the downstream innate immune signaling pathway [75]. Therefore, all these findings demonstrate that viruses acquire m^6^A in viral RNA as a strategy to evade host innate immunity. This novel mechanism is universally conserved in many RNA and DNA viruses.

In summary, we showed for the first time that RSV lacking m^6^A methylation induces higher RIG-I mediated type I IFN signaling which in turn enhances subsequent adaptive immunity. Inhibition of viral RNA m^6^A methylation may serve as a novel strategy for enhancing the immunogenicity of a vaccine virus.

## Materials and methods

### Ethics statement

The animal study was conducted in strict accordance with USDA regulations and the recommendations in the Guide for the Care and Use of Laboratory Animals of the National Research Council and was approved by The Ohio State University Institutional Animal Care and Use Committee (IACUC; animal protocol no. 2009A0221). The animals were housed within the University Laboratory Animal Resources (ULAR) facilities of The Ohio State University according to the guidelines of the Institutional Animal Care and Use Committee (IACUC). The animal care facilities at The Ohio State University are AAALAC accredited. Every effort was made to minimize potential distress, pain, or discomfort to the animals throughout all experiments.

### Cell culture and virus stock

HEp-2 (ATCC CCL-23) and A549 cells (ATCC CCL-185) were purchased from the American Type Culture Collection. A549-Dual, A549-Dual KO-*RIG-I*, A549-Dual KO-*MDA5*, and A549-Dual KO-*MAVS*-knockout cells were purchased from InvivoGen (San Diego, CA), and were supplemented with Normocin (100 μg ml^−1^), blasticidin (10 μg ml^−1^) and zeocin (100 μg ml^−1^). The wild type U2OS cells and METTL3-knockdown U2OS cell lines were generous gifts from Dr. Yang Shi (Harvard Medical School, Boston, MA). All cell lines were grown in Dulbecco’s modified Eagle’s medium (DMEM; Life Technologies, Carlsbad, CA) supplemented with 10% FBS and were confirmed free from mycoplasma by the LookOut Mycoplasma PCR Detection Kit (Sigma, St. Louis, MO). Recombinant RSV (rgRSV) strain A2 expressed GFP as the first gene, and rgRSV-ΔG was derived from rgRSV by deletion of the G gene [76,77].

### Generation of *METTL3* knockout A549 cell line

CRISPR/Cas9 was used to knock out the *METTL3* gene in A549 cells. The three sgRNAs designed for METTL3 were: sgRNA1: 5’ TCTCTTCATTCTTAGATCTA 3’, sgRNA2: GTTGGAGACAATGCTGCCTC; sgRNA3: GGGCTGTCACTACGGAAGGT. The control sgRNA was: 5’ GTACGTCGGTATAACTCCTC 3’. All sgRNAs were cloned into the lentiviral vector (plentiCRISPR v2). 293T cells (2.5×10^6^) were transfected with the lentiviral vector expressing both the sgRNA and Cas9 (5 μg), the packaging plasmid (5 μg), and the VSV-G envelope expression plasmid (2.5 μg) to generate lentiviral particles that were used to transduce A549 cells (2×10^5^). After puromycin (2 μg/ml) selection, the transduced cells were single cell cloned. The knockout/knockdown of *METTL3* expression in A549 cells were confirmed by Western blot. Genome editing of the targeted region was also confirmed by PCR of exon 4 of *METTL3* from genomic DNA followed by sequencing.

### Plasmids and site-directed mutagenesis

Plasmid (RW30) encoding the full-length antigenomic cDNA of RSV strain A2 with GFP inserted between the leader and the NS1 gene, and support plasmids expressing RSV A2 strain N protein (pTM1-N), P protein (pTM1-P), L protein (pTM1-L), and M2-1 protein (pTM1-M2-1) were generously provided by Dr. Peter Collins, (NIAID, Bethesda, MD) [78]. Mutations to the potential m^6^A sites in G gene/G gene region were introduced into the RW30 plasmids using QuikChange site-directed mutagenesis kit (Stratagene, La Jolla, CA). RW30-GALL (+) containing synonymous mutations in a total of 17 m^6^A sites in the G gene region of antigenome/G mRNA was reported previously (**S1 Fig**) [67]. Based on m^6^A-seq data, a total of 3 putative m^6^A sites were found in G gene in genome (**S2 Fig**), which were mutated using synonymous mutations. The resultant plasmid was named RW30-GALL (-). Finally, all putative m^6^A sites in the G gene region of antigenome/G mRNA and G gene of genome were mutated by combination of GALL (+) and -GALL (-), which resulted in RW30-GALL (+/-). All plasmid sequences were confirmed by DNA sequencing.

### Recovery of RSV from the full-length cDNA clones

rgRSV mutants were rescued from the full-length cDNA of the RSV A2 strain [78]. HEp-2 cells were infected with MVA-T7 at an MOI of 1.0, then transfected with 1.2 μg of plasmid RW30 or RW30 mutant, 0.4 μg of pTM1-N, 0.2 μg of pTM1-P, 0.1 μg of pTM1-M2-1, and 0.1 μg of pTM1-L using the Lipofectamine 3000 reagent (Life Technologies). At day 4 post-transfection, the cells were harvested using scrapers and were co-cultured with new flask of HEp-2 cells at 50 to 60% confluence. When an extensive cytopathic effect (CPE) was observed, the cells were subjected to three freeze-thaw cycles, followed by centrifugation at 4,000×g for 10 min. The supernatant was subsequently used to infect new HEp-2 cells. The successful recovery of the rgRSV was confirmed by the presence of green fluorescent cells, followed by RT-PCR and sequencing. Recombinants rgRSV carrying GALL(+), GALL(-), and a combination of GALL(+) and GALL(-) were designated as rgRSV- GALL(+), GALL(-), and GALL(+/-), respectively.

### Purification of rgRSV particles that are naturally defective in m^6^A

RSV particles that are naturally defective in m^6^A were purified using our established protocol [79]. Ten T150 flasks of wild type U2OS cells and *METTL3*-knockdown U2OS cells were infected with rgRSV at an MOI of 0.01. When extensive cytopathic effects (CPE) were observed, cell culture supernatants were harvested and clarified by centrifugation at 10,000 × g for 30 min. Virus was concentrated through a 35% (wt/vol) sucrose cushion by centrifugation at 30,000 × g for 2 h at 4°C in a Ty 50.2 rotor (Beckman, Brea, CA). The pellet was resuspended in NTE buffer (0.05 M Tris-HCl, 0.15 M NaCl, 15 mM CaCl2, pH 6.5). Virus was further purified through 30–50% sucrose gradient ultracentrifugation. The band containing virus particles were collected and centrifuged at 25,000 × g for 2 h at 4°C. The pellet was resuspended in DMEM with 10% trehalose.

### Purification of rgRSV mutants

Recombinant RSV containing m^6^A mutations on antigenome [rgRSV-GALL(+)), genome (rgRSV-GALL (-)], both genome and antigenome (rgRSV-GALL (+/-), and rgRSV with G gene deletion mutant (rgRSV-ΔG) were grown on 10 T150 flasks of HEp-2 cells. RSV virions were purified using the procedure described above.

### Viral RNA extraction and real-time RT-PCR

Virion RNA was isolated from the purified virions using TRIzol reagent (Life Technologies, Carlsbad, CA) following the manufacturer’s instruction. Where indicated, total RNA was extracted from virus-infected cells using TRIzol reagent. Viral genome and antigenome RNA copies were quantified using reverse transcript (RT) real-time PCR using TB-Green premix Ex Taq (TaKaRa, Kusatsu, Shiga Prefecture, Japan).

### Colorimetric quantification of viral m^6^A methylation

Virion RNA was extracted from viruses purified by sucrose cushion ultracentrifugation described above. Total m^6^A modification level of virion RNA was quantified by m^6^A RNA Methylation Assay Kit (Abcam, ab185912) based on the manufacturer’s instruction [79]. Briefly, each amount of virion RNA was bound to strip wells using a RNA high binding solution, and m^6^A was detected using a specific capture anti-m^6^A antibody and then quantified colorimetrically by reading the absorbance in a microplate spectrophotometer at a wavelength of 450 nm. A standard curve was generated using known m^6^A methylated RNA (range from 0.02 to 1 ng of m^6^A) as a positive control. The m^6^A content was calculated from each RNA sample based on their OD450 values. The percent change was calculated by dividing m^6^A levels in virion RNA from U2OS KD cells by those from the wt U2OS cells. The m^6^A level of rgRSV mutants grown in HEp-2 cells was calculated using the parental rgRSV grown in HEp-2 cells.

### Measurement of interferon

For viral RNA transfection, same copies of viral RNA were transfected into regular A549 or A549 dual KO cells using Lipofectamine 3000 (ThermoFisher Scientific, Waltham, MA). At different time points after transfection, culture medium was harvested for quantification of IFN-β by commercial ELISA following the manufacturer’s instruction. Known concentrations of human IFN-β were used to generate the standard curve. For viral infection, A549 cells were infected by equal MOIs of each virus treated or untreated with cycloheximide (CHX), cell lysates were collected at indicated time points and total RNA was extracted by Trizol reagent following the manufacturer’s instruction. IFN mRNA was quantified by real-time RT-PCR and normalized with mRNA of β-actin.

### Antibodies and Western blotting

The antibodies used in this study were anti-METTL3 (Proteintech, 15073-1-AP), anti-RIG-I (Abcam, ab180675), anti-MDA5 (Abcam, ab79055), anti-IRF3 (Phospho S386) (Abcam, ab76493), anti-IRF3 (Abcam, ab25950), anti-YTHDF2 (abcam, ab220163), anti-actin (Proteintech, 66009). For viral RNA transfection, equal copies of viral RNA at several dilutions were transfected into regular A549 or A549 dual KO cells using Lipofectamine 3000 (ThermoFisher Scientific, Waltham, MA). Cells were harvested and lysed in 1×RIPA buffer (Abcam, ab156034). Western blot, protein expression was measured by using antibody against RIG-I, MDA5, IRF3 and IRF3p (S386). β-actin was used as the loading control.

### RIG-I ubiquitination assay

Ubiquitination assay was performed as previously described [51, 79]. Specifically, purified RIG-I (1.0 mM) was first incubated with purified RSV virion RNA (ranging from 0.1 to 150 ng/μl) in buffer A (20 mM HEPES, pH 7.5, 150mM NaCl, 1.5mM MgCl2, 2mM ATP and 5mM DTT) at RT for 15 min. The RIG-I: RNA complex (to the final RIG-I concentration of 0.5 mM) was then further incubated with 20 mM ubiquitin, 1 mM mE1, 5 mM Ubiquitin-conjugating enzyme E2 13(Ubc13), 2.5 mM Ubiquitin-conjugating enzyme E2 variant 1A (Uev1A) and 0.25 mM E3 RIPLET in buffer B (50 mM Tris pH 7.5, 10 mM MgCl2, 5 mM ATP and 0.6 mM DTT) at 37°C for 30 min. Reactions were quenched with SDS loading buffer, boiled at 96°C for 5 min, and analyzed on SDS-PAGE by anti-RIG-I or anti-Ub Western blot. The density of each lane was quantified by Image J. The ratio between m^6^A-deficient virion RNA and wild type virion RNA at each RNA concentration was calculated based on the density of ubiquitination bands.

### Viral replication kinetics

RSV replication kinetics was determined as described previously [46]. Confluent A549 cells or KO A549 cells in 12-well-plates were infected with rgRSV or mutant rgRSV at an MOI of 0.1. After 1 h of adsorption, the inoculum was removed and the cells were washed three times with DMEM. Fresh DMEM (supplemented with 2% FBS) was added, and the infected cells were incubated at 37°C. At different time points post-inoculation, the supernatant and cells were harvested by three freeze-thaw cycles, followed by centrifugation at 1,500 × *g* at 4°C for 15 min. The virus titer was determined by TCID_50_ assay in HEp-2 cells.

### Examination of GFP expression by microscope and flow cytometry

A549 or HEp-2 cells were infected with rgRSV or mutants at an MOI of 0.1, and GFP expression was monitored at the indicated times by fluorescence microscopy. At the indicated time points, cells were trypsinized and fixed in 0.01% of paraformaldehyde solution and the number of GFP-positive cells quantified by flow cytometry.

### Analysis of RSV gene expression and RNA replication

Confluent A549 cells were infected with rgRSV and rgRSV mutants at an MOI of 0.1. At 18, 24, and 48h postinfection, the cell culture supernatant was removed and the cells were lysed in 150 μl of RIPA buffer (Abcam) for Western blot analysis using RSV serum antibody (Virostat), F (Abcam) or anti-β-actin antibody (Proteintech). For analysis of RNA replication and mRNA synthesis, total RNA was extracted at 18, 24, and 48h postinfection, and RSV genome and G mRNA was quantified by real-time RT-PCR.

### Replication and spreading of RSV in HBE culture

Purified virions were titrated on HEp-2 cells and were diluted in HBE cell medium. The apical surface of well-differentiated HBE cells in Transwells was washed with PBS for 2 h and the basal medium was changed before adding the virus (400 TCID_50_) was added to the apical surface of the cultures as described previously [46]. Fluorescent cells were visualized with an EVOS2 fl inverted fluorescence microscope (Life Technologies, Carlsbad, CA). At the indicated times, 100 μl of PBS was added to the apical surface of HBE culture, gently rocked for 30 min, and collected for virus titration by TCID_50_ assay. The amount of the HBE culture expressing GFP-positive cells was determined from a digital image using Image J Software.

### Innate immune responses of m^6^A-deficient rgRSV mutants in mice

Twenty-five 4-week old BALB/c mice were grouped randomly into 5 groups, which were intranasally inoculated with 20 μl of PBS or PBS containing 5×10^5^ TCID_50_ of rgRSV, rgRSV-GALL(+), rgRSV-GALL(-), or rgRSV-GALL(+/-). After inoculation, clinical signs were scored. At 48h post-inoculation, all mice were euthanized, and the whole lung was homogenized in 500μL of PBS. Total RNA was extracted from 150μL of this lysate and dissolved in 50μL RNase-free water. Reverse transcription (RT) was carried out using 2μL of total RNA and Oligo (dT)23 in 20μL system. The synthesized cDNA was 1:2 diluted and used for real-time PCR to quantify mouse cytokines including IFN-β (type I), IFN-γ (type II), IFN-λ (type III), IL-6, and TNF-α. Primers used for qPCR were listed in **S1 Table**.

### Immunogenicity of m^6^A-deficient rgRSV mutants in mice

Twenty-five 4 to 6-week-old specific-pathogen-free (SPF) female BALB/c mice (Charles River Laboratories, Wilmington, MA) were randomly divided into 5 groups (5 mice per group). Mice in group 1 to 5 were intranasally inoculated with 1.0×10^6^ TCID_50_ of rgRSV-GALL(+), rgRSV-GALL(-), rgRSV-GALL(+/-), parental rgRSV, and PBS, respectively. After inoculation, the animals were evaluated daily for mortality, body weight change, and the presence of any symptoms of RSV infection. The severity of clinical signs associated with RSV infection was scored based on the following criteria: grade 3 (severe) was characterized by ruffled fur, hyperexcitability, tremors, and circling; grade 2 (moderate) was characterized by ruffled fur, hunched posture, and reduced food and water intake; grade 1 (mild) was characterized by mild ruffled fur; grade 0 was defined as no symptoms. Blood samples were collected from each mouse weekly by orbital sinus blood sampling for neutralizing antibody detection.

### Replication and immunogenicity of rgRSV in cotton rats

For assessing the replication of each recombinant RSV in cotton rats, twenty 6-week-old specific-pathogen-free (SPF) male cotton rats (Envigo, Indianapolis, IN) were randomly divided into 4 groups (5 cotton rats per group). Groups 1 to 4 were intranasally inoculated with 2.0×10^5^ TCID_50_ of rgRSV-GALL(+), rgRSV-GALL(-), rgRSV-GALL(+/-), and parental rgRSV, respectively. At day 4 post-infection, the cotton rats were sacrificed, and left lungs and nasal turbinate were collected for both virus isolation and histological analysis. The right lung was fixed in 4% formaldehyde for histology.

For the immunogenicity evaluation, nine groups of cotton rats (5 per group) were included. Groups 1 to 4 were intranasally inoculated with 2.0×10^5^ TCID_50_ of rgRSV-GALL(+), rgRSV-GALL(-), rgRSV-GALL(+/-), and parental rgRSV, respectively. Groups 5–8 were intranasally inoculated with 2.0×10^3^ TCID_50_ of each virus. Group 9 was inoculated with PBS and served as unimmunized challenged control. All groups were evaluated daily for any possible abnormal reaction and blood was collected by orbital sinus bleeding at weeks 2, 3, and 4 for measurement of neutralizing antibody. At week 4 post-immunization, cotton rats in all groups were challenged with 2.0×10^5^ TCID_50_ of parental rgRSV via the intranasal route. At day 4 post-challenge, all cotton rats were sacrificed and their left lung and nasal turbinate were collected for virus titration as described previously. In addition, the right lung was fixed in 4% formaldehyde for histology.

### Determination of viral titer in lung and nasal turbinate

The nasal turbinate and the left lung from each cotton rat were removed, weighed, and homogenized in either 3 ml or 2 ml of DMEM. The lung was homogenized using a Precellys 24 tissue homogenizer (Bertin instruments, Rockville, MD) by following the manufacturer’s recommendations. The mucosa from the nasal turbinates was homogenized by hand with a 0.90mm CoorsTek mortar and pestle (Golden, CO) and sterile sand. The presence of infectious virus was determined by TCID_50_ assay in HEp-2 cells[46].

### Determination of RSV-neutralizing antibody

RSV-specific neutralizing antibody titers were determined using a plaque reduction neutralization assay [46,80]. Briefly, mouse or cotton rat sera were collected by orbital sinus blood sampling weekly until challenge. The serum samples were heat inactivated at 56°C for 30 min. Twofold dilutions of the serum samples were mixed with an equal volume of DMEM containing approximately 50 TCID_50_/well rgRSV in a 96-well plate, and the plate was incubated at room temperature for 1 h with constant rotation. The mixtures were then transferred to confluent HEp-2 cells in a 96-well plate in triplicate. After 1 h of incubation at 37°C, the virus-serum mixtures were removed and the cells were overlaid with 0.75% methylcellulose in overlay media (1× MEM, 2% FBS, Sodium bicarbonate, 25mM HEPES, 1% L-Glutamine, 1% Pen Strep) and incubated for another 3 days before counting the fluorescent foci. The numbers of foci at each serum dilution were plotted and the 50% plaque reduction titer was used as the RSV-specific neutralizing antibody titer.

### Analysis of RSV-specific T cell immune responses

To measure RSV F-specific T cell immune response, spleens were collected at week 4 post-immunization, spleen cells were isolated and prepared as previously described. The cell concentrations were adjusted to 3 × 10^6^ cells/mL and 100 μl were added into each well (3 wells per spleen sample) of a 96-well microtiter plate and cultured either alone or in the presence of 50 μg/ml of RSV pre-fusion F protein (kindly provided by Dr. Mark Peeples at Nationwide Children’s Hospital, Columbus, OH) for 5 days at 37°C in a 5% CO2 atmosphere. Culture supernatants were collected from each well and frozen at −80°C until analysis of secreted cytokines using the Bio-Plex Pro Mouse Cytokine Standard 23-Plex, Group I (Bio-Rad Laboratories Inc, Hercules, CA) per manufacturer’s instructions. The antibodies used for staining were summarized in **S2 Table**. The frequencies of RSV-specific Th1 (IFN-λ+CD4+CD3+ and TNF-α, +CD4+CD3+), Th2 cells (IL-4+CD4+CD3+, IL-5+CD4+CD3+, IL-10+CD4+CD3+), Th17 (IL-17A+ CD4+ CD3+), and Tfh (IL-21+ CD4+ CD3+) cells were determined by intracellular staining with the corresponding anti-cytokine Abs (at a dilution of 1:5000) after additional incubation in the presence of PMA and ionomycin. The cells were then analyzed with the aid of an Attune flow cytometer and data were expressed as mean % positive cells ± standard deviation.

### Pulmonary histology

After sacrifice, the right lung of each animal was removed, inflated, and fixed with 4% neutral buffered formaldehyde. Fixed tissues were embedded in paraffin and a microtome used to generate 5 μm sections. Slides were then stained with hematoxylin-eosin (H&E) for the examination of histological changes by light microscopy. Histopathological changes were evaluated based on the extent of interstitial inflammation, edema, alveolitis, bronchiolitis, and peribronchiolar inflammation [80].

### Statistical analysis

All data and statistical analysis were performed by using GraphPad Prism 8.0 (GraphPad Software, San Diego, CA). Data were analyzed using unpaired Student’s t-test and a *P* value of <0.05 was considered statistically significant. *P < 0.05; **P < 0.01; ***P < 0.001; ****P < 0.0001.

## Supporting information

S1 Fig Mutagenesis strategy in putative m^6^A sites in the RSV G gene region in the antigenome. Schematic diagram of the RSV genome is shown. A total of 17 putative m^6^A sites in the G gene region are highlighted by green. These 17 m^6^A sites were mutated using synonymous mutations by altering the critical A or C residues in the m^6^A motifs to produce rgRSV-GALL(+) which lacks all putative m^6^A modification sites in the G gene region in the antigenome. Consensus m^6^A motifs (green) and inactivating mutations (red) are shown. Dashes represent nucleotides not shown. G gene sequence of RSV A2 strain (accession number M74568) is shown.
(TIFF)

S2 Fig Mutagenesis strategy in putative m^6^A sites in the RSV G gene in the genome. A total of 3 putative m^6^A sites in the G gene are highlighted by green. These 3 m^6^A sites were mutated using synonymous mutations by altering the critical A or C residues in the m^6^A motifs to produce rgRSV-GALL(-) which lacks all putative m^6^A modification sites in the G gene in the genome.
(TIFF)

S3 Fig m^6^A-deficient RSV RNA enhances expression of molecules involved in type I IFN signaling pathway. Confluent A549 cells were transfected with virion RNA of RSV grown on *METTL3* KD U2OS or WT U2OS cells at doses of 1.0×10^9^, 2×10^8^ and 4×10^7^ RNA copies. Poly I:C was used as a positive control. At 24, 48 and 72h post-transfection, cell lysates were analyzed by Western blotting using antibodies specific to RIG-I, MDA5, IRF3, IRF3 (phosphorylated at S386) or β-actin.
(TIF)

S4 Fig Affinity binding assay of RIG-I with RSV RNA. (A) Western blot of pulldown RIG-I showing the equal pulldown efficiency for rgRSV-GALL(+/-) and rgRSV-WT RNA. (B) Western blot of pulldown RIG-I showing the equal pulldown efficiency for virion RNA from rgRSV particles grown in *METTL3*-KD and METTL3-WT. Antigenome (C) and genome (D) of rgRSV-GALL(+/-) and rgRSV-WT pulled down by RIG-I were quantified by real-time RT-PCR. Antigenome (E) and genome (F) of rgRSV grown in *METTL3*-KD and WT U2OS cells pulled down by RIG-I were quantified by real-time RT-PCR. Error bars represent SD from *n*  =  3 independent experiments. *P<0.5, **P < 0.01, ***P < 0.001, ****P < 0.0001.
(TIF)

S5 Fig In vitro ubiquitination analysis of RIG-I. (A) Ubiquitination analysis of RIG-I at a low RNA concentration. 1.0 μM of purified RIG-I were incubated with 1ng/μl of 42 bp dsRNA or 2.0 ng/ μl of virion RNA from rgRSV-GALL(+/-), parental rgRSV, *METTL3*-KD U2OS cells, or wt U2OS cells with or without RIPLET. Ubiquitination of RIG-I was analyzed by anti-RIG-I blot. (B) Virion RNA from *METTL3*-KD U2OS cells enhances RIG-I ubiquitination compared to virion RNA from wt U2OS cells at RNA concentrations of 54 and 150 ng/ μl. 1.0 μM of purified RIG-I was incubated with 1ng/μl of 42 bp dsRNA or different doses (54 and 150 ng/μl) of virion RNA from *METTL3*-KD U2OS cells or wt U2OS cells with or without RIPLET. (C) rgRSV-GALL(+/-) virion RNA enhances RIG-I ubiquitination compared to rgRSV-GALL(+/-) virion RNA at concentrations of 50 and 150 ng/μl. 1.0 μM of purified RIG-I were incubated with 1ng/μl of 42 bp dsRNA or different doses (50 and 150 ng/μl) of virion RNA from rgRSV-GALL(+/-) or parental rgRSV with or without RIPLET. The density of each lane was quantified by Image J. The length of lane used for quantification was indicated by the line in the left side. The ratio between m^6^A-deficient virion RNA and wild type virion RNA at each RNA concentration was indicated.
(TIF)

S6 Fig A proposed model of how m^6^A-deficient RSV modulates innate and adaptive immunity. Upon entry of RSV particles in cells, m^6^A-deficient RSV genome and antigenome are more easily detected by RIG-I, transmitting a stronger signaling to the adaptor protein MAVS, which leads to a higher phosphorylation of IRF-3 by TBK1/inducible I κB kinase (IKK-*i*). The IRF-3 homodimers and/or heterodimers are formed and translocated into the nucleus, resulting in a higher expression of type I IFNs and perhaps other cytokines and chemokines. A higher innate immunity will likely stimulate antigen presenting cells (such as macrophage and dendritic cells) that mediate the cellular immune response by processing and presenting antigens for recognition by T cells. Interferon can also directly stimulate T and B cells which lead to a stronger cellular and humoral immunity.
(TIFF)

S1 Table Primers used for quantification of mouse cytokines by real-time RT-PCR. (DOCX)

S2 Table Antibodies used for RSV F-specific T cell response. (DOCX)
