# Supplementary figures and images for: Viral RNA N6-methyladenosine modification modulates both innate and adaptive immune responses of human respiratory syncytial virus
Source: PLoS Pathog. 2021 Dec 20;17(12):e1010142. doi: 10.1371/journal.ppat.1010142 (PMC8759664; doi:10.1371/journal.ppat.1010142)

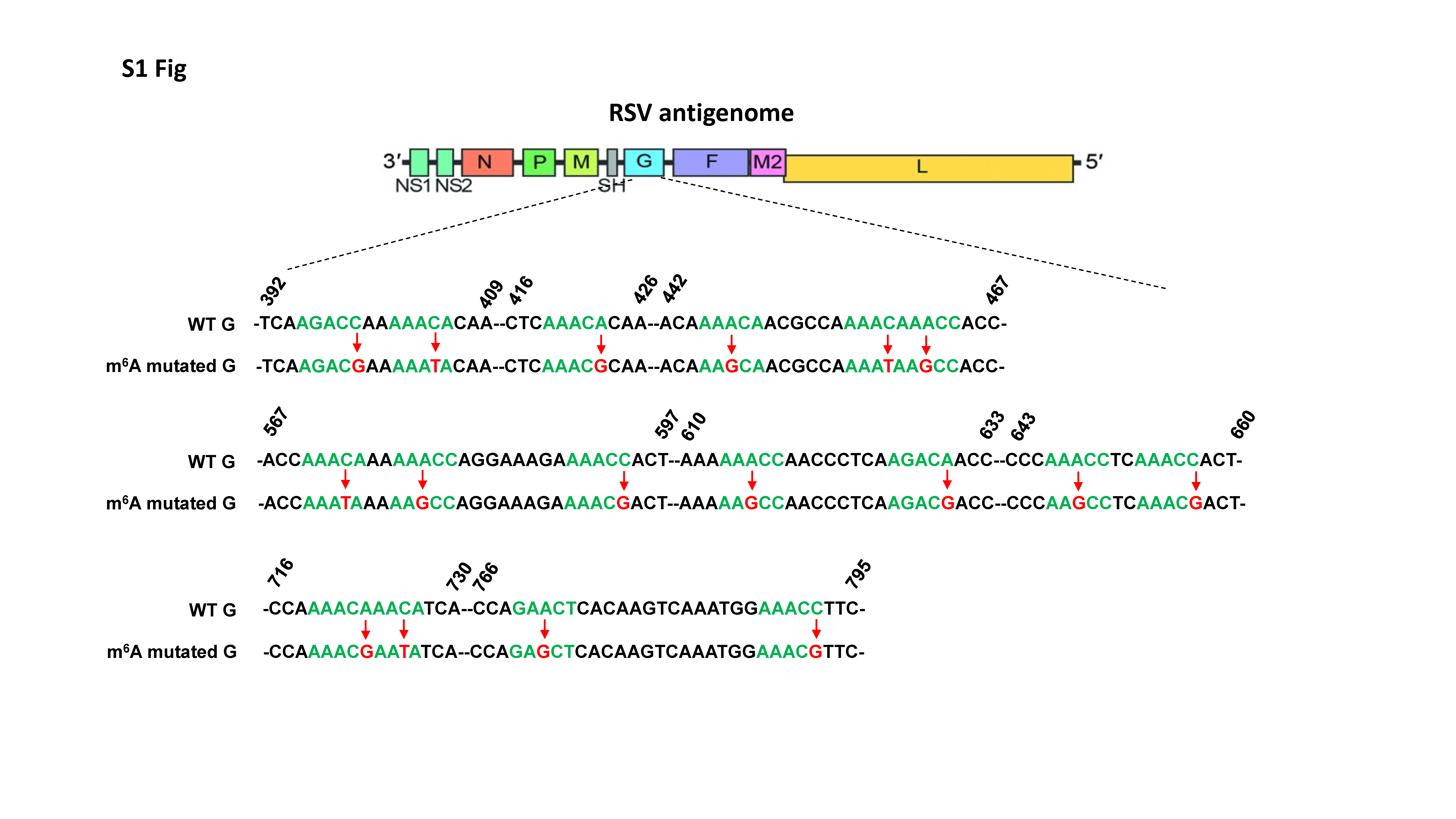

Supplement: S1 Fig — Schematic diagram of the RSV genome is shown. A total of 17 putative m6A sites in the G gene region are highlighted by green. These 17 m6A sites were mutated using synonymous mutations by altering the critical A or C residues in the m6A motifs to produce rgRSV-GALL(+) which lacks all putative m6A modification sites in the G gene region in the antigenome. Consensus m6A motifs (green) and inactivating mutations (red) are shown. Dashes represent nucleotides not shown. G gene sequence of RSV A2 strain (accession number M74568) is shown. (TIFF) [file ppat.1010142.s001.tiff]

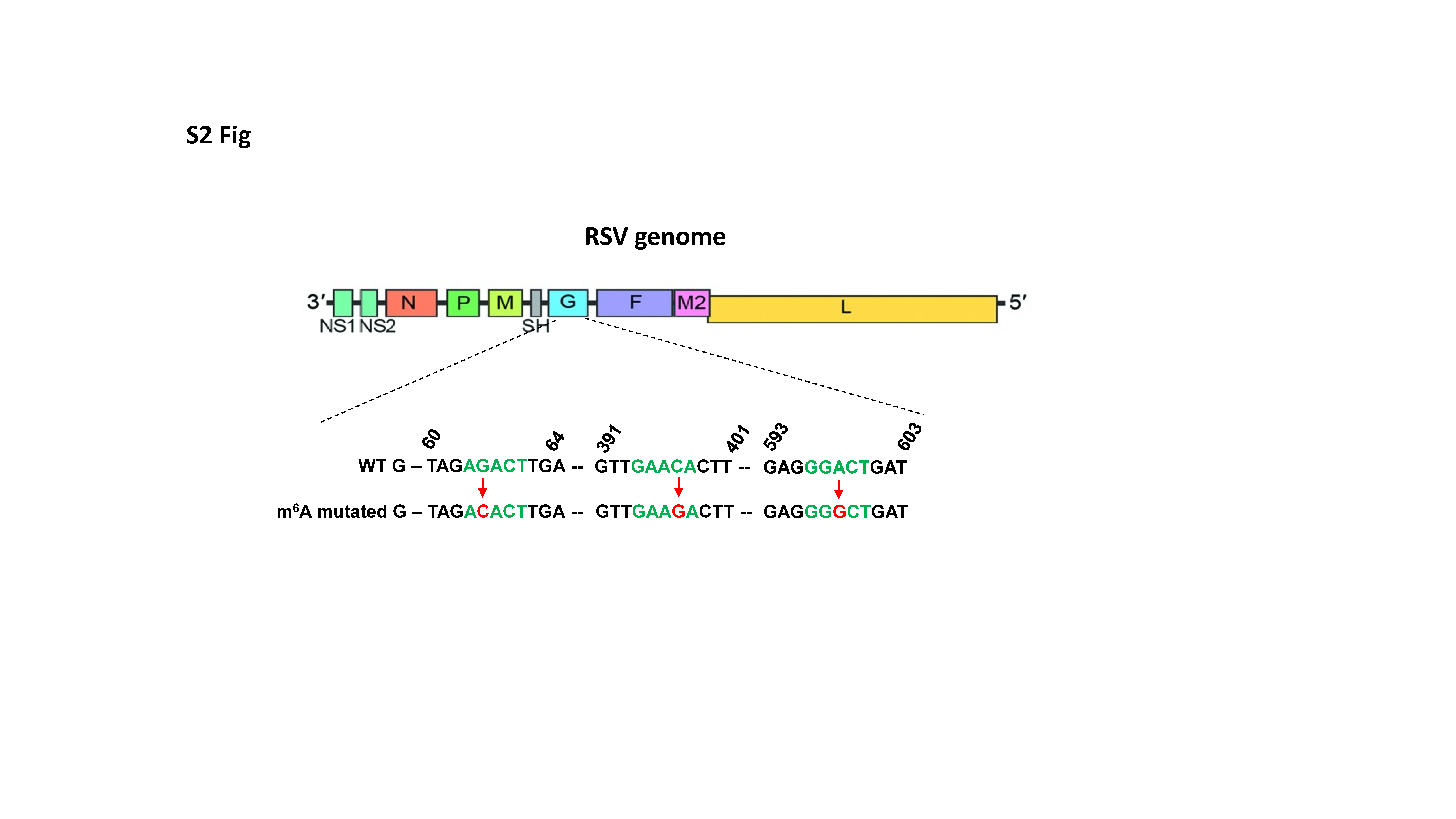

Supplement: S2 Fig — A total of 3 putative m6A sites in the G gene are highlighted by green. These 3 m6A sites were mutated using synonymous mutations by altering the critical A or C residues in the m6A motifs to produce rgRSV-GALL(-) which lacks all putative m6A modification sites in the G gene in the genome. (TIFF) [file ppat.1010142.s002.tiff]

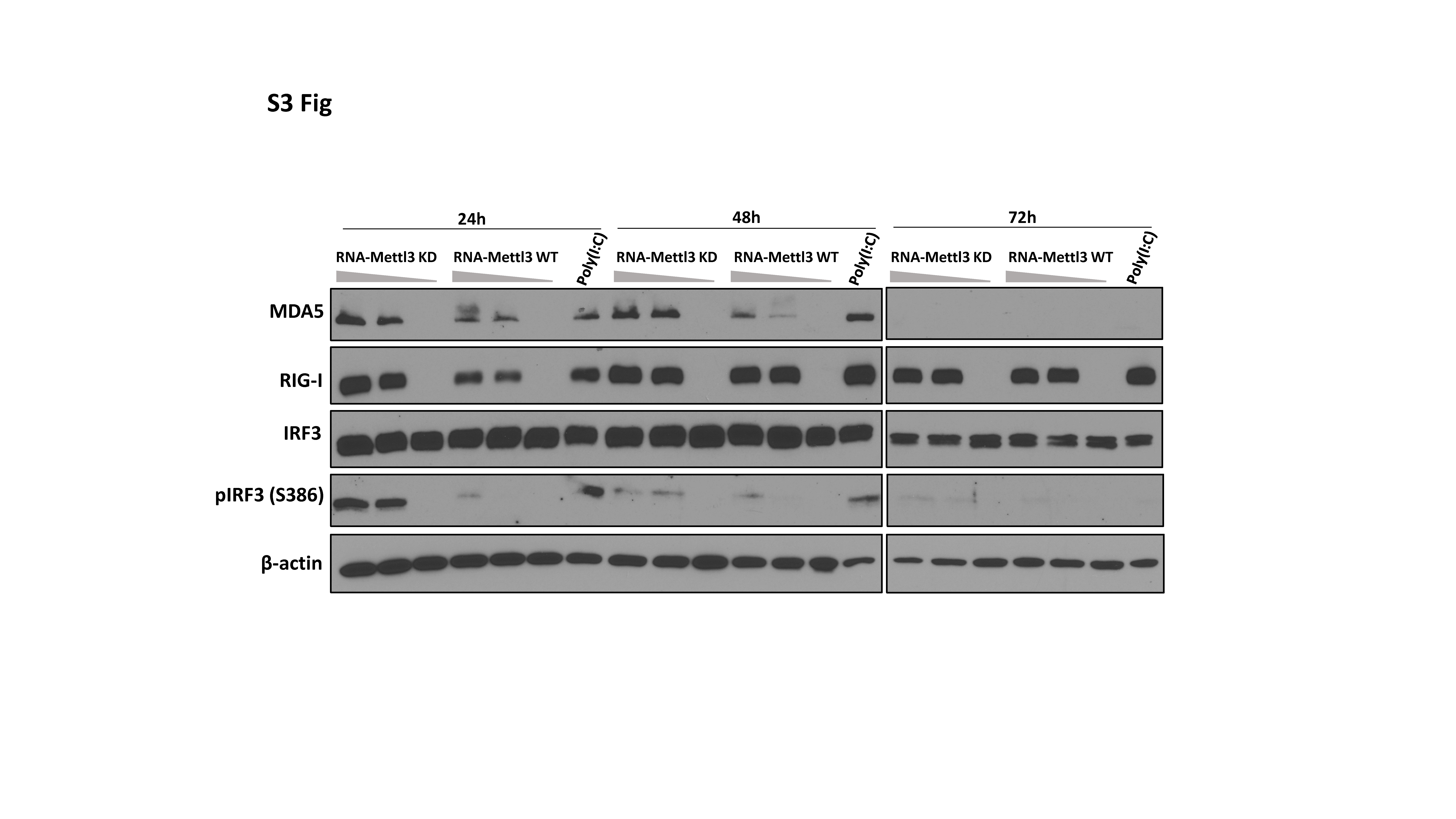

Supplement: S3 Fig — Confluent A549 cells were transfected with virion RNA of RSV grown on METTL3 KD U2OS or WT U2OS cells at doses of 1.0×10^9^, 2×10^8^ and 4×10^7^ RNA copies. Poly I:C was used as a positive control. At 24, 48 and 72h post-transfection, cell lysates were analyzed by Western blotting using antibodies specific to RIG-I, MDA5, IRF3, IRF3 (phosphorylated at S386) or β-actin. (TIF) [file ppat.1010142.s003.tif]

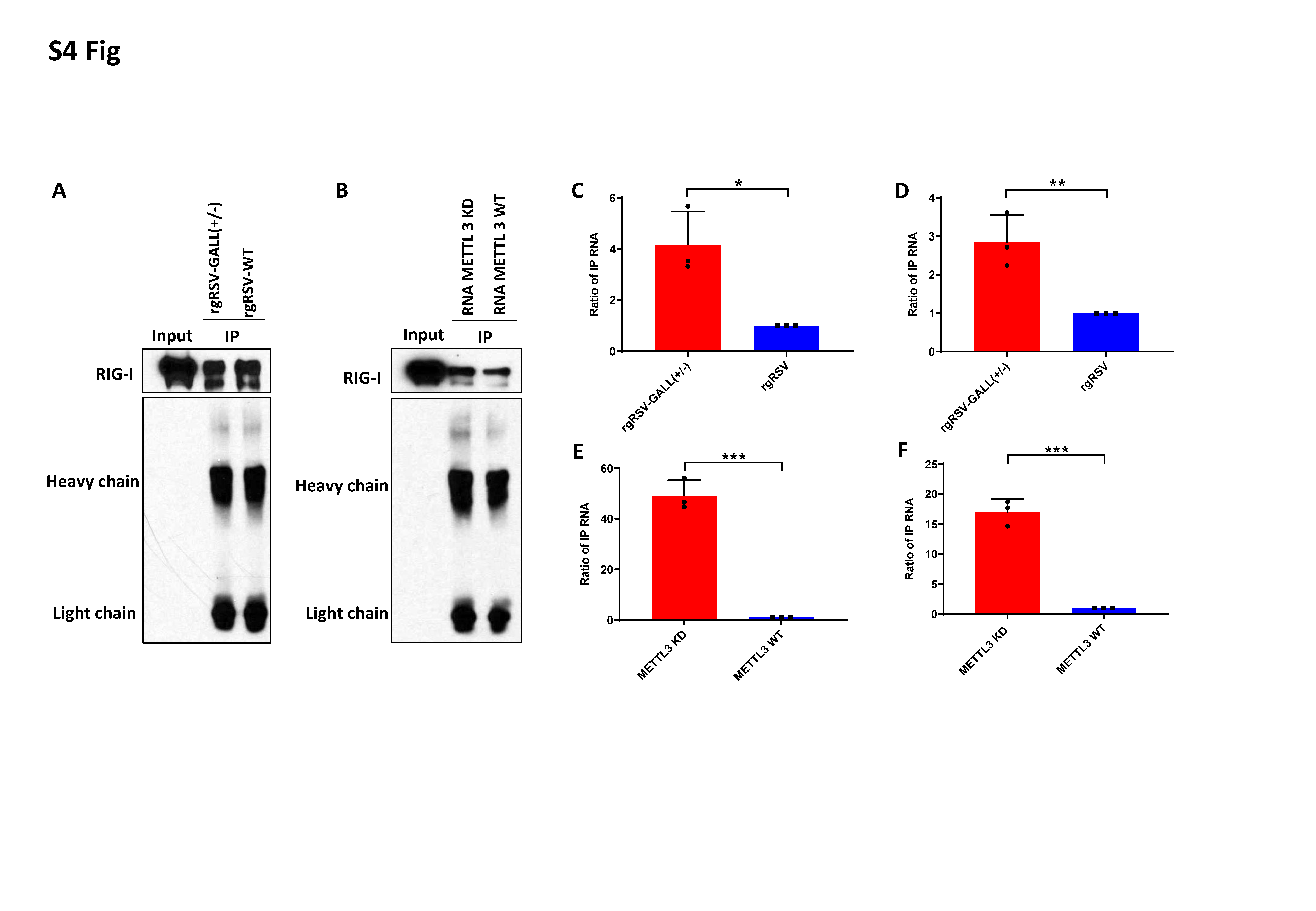

Supplement: S4 Fig — (A) Western blot of pulldown RIG-I showing the equal pulldown efficiency for rgRSV-GALL(+/-) and rgRSV-WT RNA. (B) Western blot of pulldown RIG-I showing the equal pulldown efficiency for virion RNA from rgRSV particles grown in METTL3-KD and METTL3-WT. Antigenome (C) and genome (D) of rgRSV-GALL(+/-) and rgRSV-WT pulled down by RIG-I were quantified by real-time RT-PCR. Antigenome (E) and genome (F) of rgRSV grown in METTL3-KD and WT U2OS cells pulled down by RIG-I were quantified by real-time RT-PCR. Error bars represent SD from n = 3 independent experiments. *P<0.5, **P < 0.01, ***P < 0.001, ****P < 0.0001. (TIF) [file ppat.1010142.s004.tif]

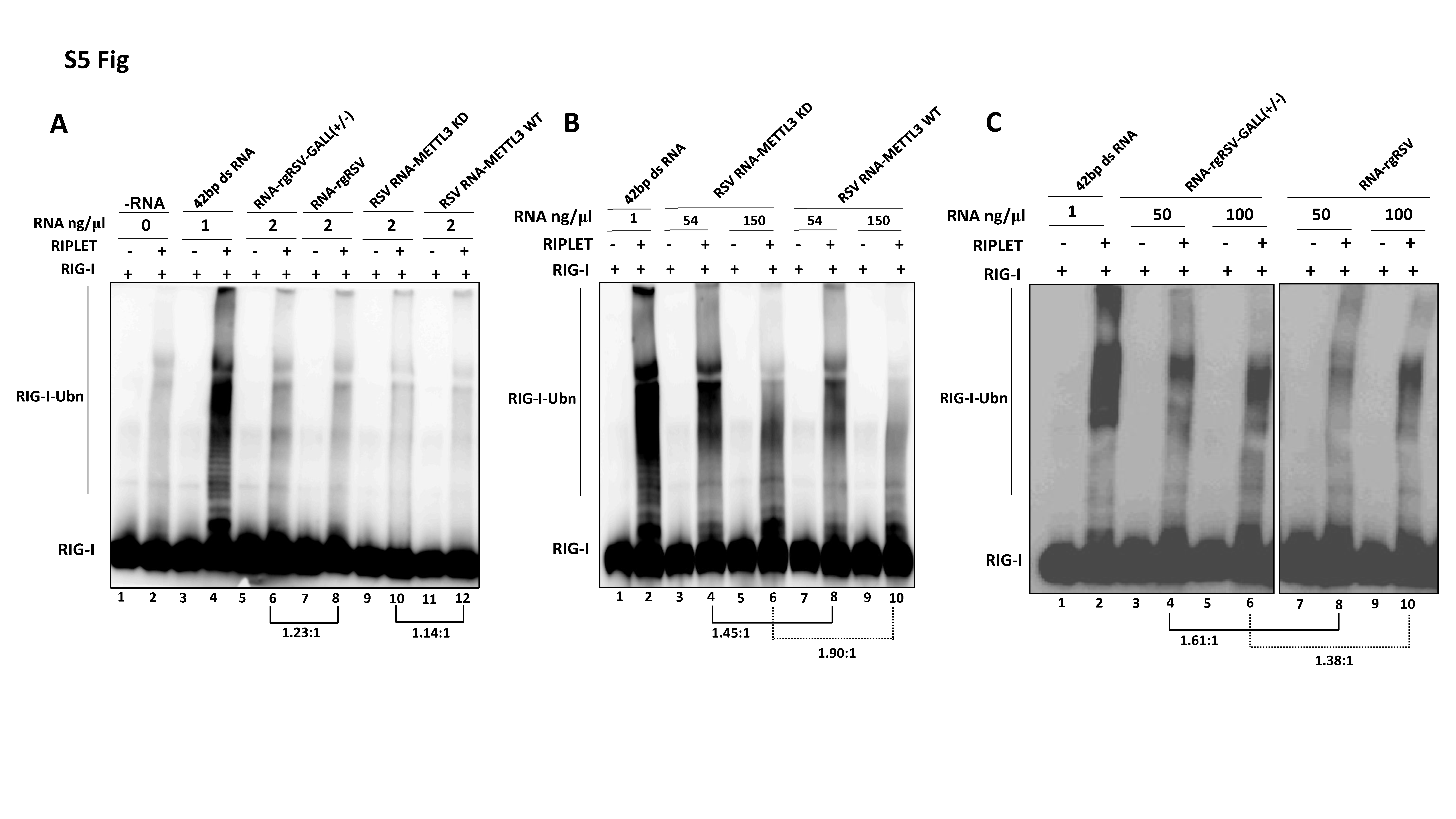

Supplement: S5 Fig — (A) Ubiquitination analysis of RIG-I at a low RNA concentration. 1.0 μM of purified RIG-I were incubated with 1ng/μl of 42 bp dsRNA or 2.0 ng/ μl of virion RNA from rgRSV-GALL(+/-), parental rgRSV, METTL3-KD U2OS cells, or wt U2OS cells with or without RIPLET. Ubiquitination of RIG-I was analyzed by anti-RIG-I blot. (B) Virion RNA from METTL3-KD U2OS cells enhances RIG-I ubiquitination compared to virion RNA from wt U2OS cells at RNA concentrations of 54 and 150 ng/ μl. 1.0 μM of purified RIG-I was incubated with 1ng/μl of 42 bp dsRNA or different doses (54 and 150 ng/μl) of virion RNA from METTL3-KD U2OS cells or wt U2OS cells with or without RIPLET. (C) rgRSV-GALL(+/-) virion RNA enhances RIG-I ubiquitination compared to rgRSV-GALL(+/-) virion RNA at concentrations of 50 and 150 ng/μl. 1.0 μM of purified RIG-I were incubated with 1ng/μl of 42 bp dsRNA or different doses (50 and 150 ng/μl) of virion RNA from rgRSV-GALL(+/-) or parental rgRSV with or without RIPLET. The density of each lane was quantified by Image J. The length of lane used for quantification was indicated by the line in the left side. The ratio between m6A-deficient virion RNA and wild type virion RNA at each RNA concentration was indicated. (TIF) [file ppat.1010142.s005.tif]

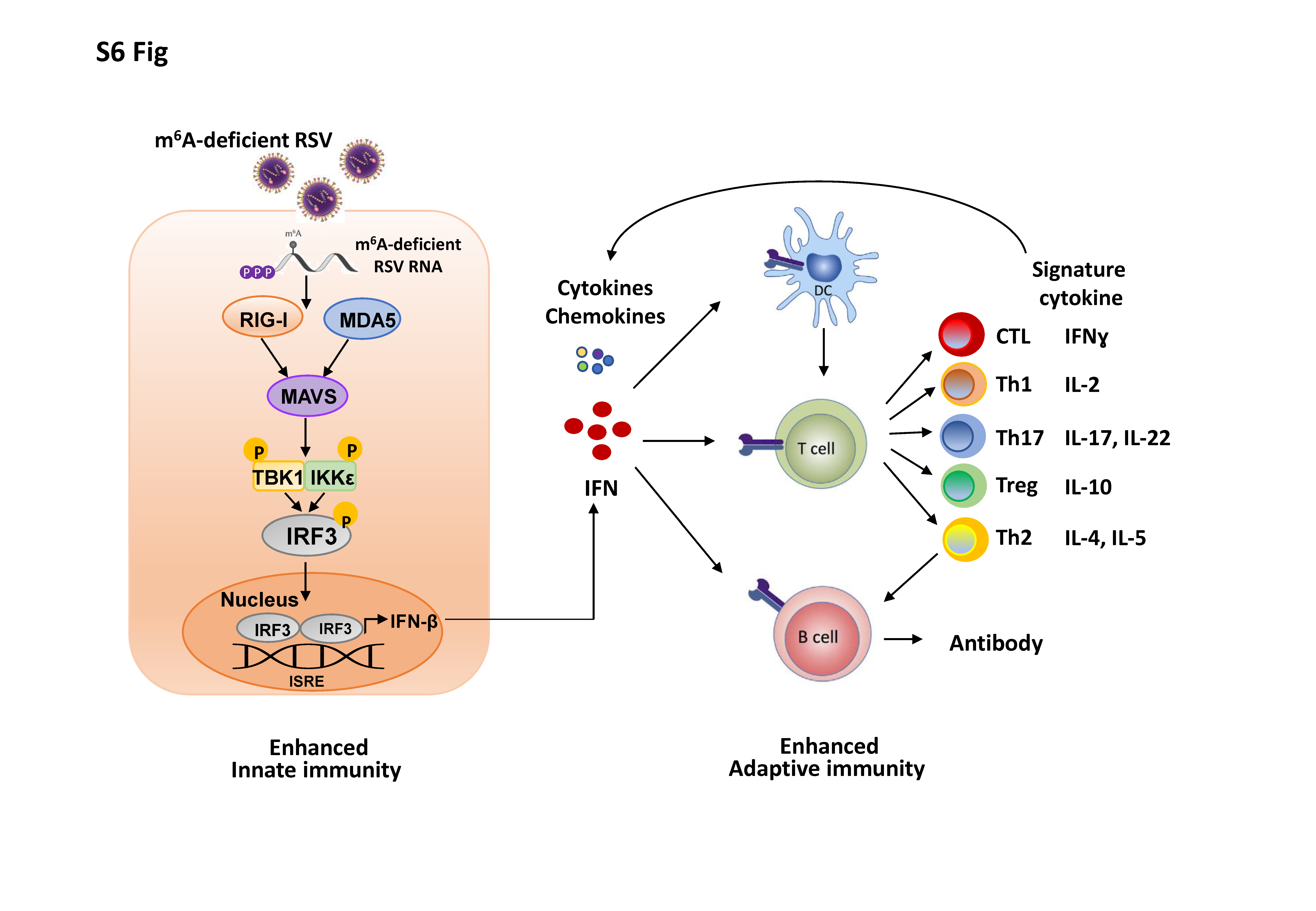

Supplement: S6 Fig — Upon entry of RSV particles in cells, m6A-deficient RSV genome and antigenome are more easily detected by RIG-I, transmitting a stronger signaling to the adaptor protein MAVS, which leads to a higher phosphorylation of IRF-3 by TBK1/inducible I κB kinase (IKK-i). The IRF-3 homodimers and/or heterodimers are formed and translocated into the nucleus, resulting in a higher expression of type I IFNs and perhaps other cytokines and chemokines. A higher innate immunity will likely stimulate antigen presenting cells (such as macrophage and dendritic cells) that mediate the cellular immune response by processing and presenting antigens for recognition by T cells. Interferon can also directly stimulate T and B cells which lead to a stronger cellular and humoral immunity. (TIFF) [file ppat.1010142.s006.tiff]
