# Supplementary material for: Viral RNA N6-methyladenosine modification modulates both innate and adaptive immune responses of human respiratory syncytial virus
Source: PLoS Pathog. 2021 Dec 20;17(12):e1010142. doi: 10.1371/journal.ppat.1010142 (PMC8759664; doi:10.1371/journal.ppat.1010142)
Supplement: S1 Table — (DOCX) [file ppat.1010142.s007.docx]

**S1 Table: Primers used for quantification of mouse cytokines by RT-qPCR**

| **Primers** | **Sequence** |
| --- | --- |
| **mIFN-beta1** | 5’- CCCTATGGAGATGACGGAGA -3’  5’- CTGTCTGCTGGTGGAGTTCA -3’ |
| **mIFN-gamma** | 5’- ACTGGCAAAAGGATGGTGACA -3’  5’- TGAGCTCATTGAATGCTTGG -3’ |
| **mIFN-lambda (subtype 2 and 3)** | 5’- GCAGCTGCAGGTCCAAGAGC -3’  5’- CTGTGGCCTGAAGCTGTGTA -3’ |
| **mIL6** | 5’- AGTTGCCTTCTTGGGACTGA -3’  5’- TCCACGATTTCCCAGAGAAC -3’ |
| **mTNF-alpha** | 5’- CGTCAGCCGATTTGCTATCT -3’  5’- CGGACTCCGCAAAGTCTAAG -3’ |
