# Supplementary material for: Viral RNA N6-methyladenosine modification modulates both innate and adaptive immune responses of human respiratory syncytial virus
Source: PLoS Pathog. 2021 Dec 20;17(12):e1010142. doi: 10.1371/journal.ppat.1010142 (PMC8759664; doi:10.1371/journal.ppat.1010142)
Supplement: S2 Table — (DOCX) [file ppat.1010142.s008.docx]

**S2 Table. Antibodies used for RSV F-specific T cell response**

| **Abs** | **Flourochrome** | **Colon#** | **Concentration** | **Catalog #** | **Company** |
| --- | --- | --- | --- | --- | --- |
| Anti-CD3 | Alexa Fluor 700 | 17A2 | 0.5mg/ml | 100216 | Biolegend |
| Anti-CD4 | Alexa Fluor 750 | GK1.5 | 0.2mg/ml | 100460 | Biolegend |
| Anti-IFNγ | Alexa Fluor 488 | XMG1.2 | 0.5mg/ml | 505813 | Biolegend |
| Anti-TNFα | PerCP Cy5.5 | MP6-XT22 | 0.2mg/ml | 506322 | Biolegend |
| Anti- IL-5 | PE | TRFK5 | 0.2mg/ml | 504304 | Biolegend |
| Anti- IL-21 | Alexa Fluor 647 | IC594R | 0.2mg/ml | 1506741 | R&D System |
| Anti- IL-10 | PE-Cy7 | JES5-16E3 | 0.2mg/ml | 505026 | Biolegend |
| Anti- IL-17A | Brilliant Violet 650 | TC11-18H10.1 | 0.5mg/ml | 506929 | Biolegend |
| Anti- IL-4 | Brilliant Violet 605 | 11B11 | 0.2mg/ml | 504125 | Biolegend |
